# A Polymer‐Assisted Spinodal Decomposition Strategy toward Interconnected Porous Sodium Super Ionic Conductor‐Structured Polyanion‐Type Materials and Their Application as a High‐Power Sodium‐Ion Battery Cathode

**DOI:** 10.1002/advs.202004943

**Published:** 2021-03-20

**Authors:** Hailong Xiong, Ruicheng Qian, Zhilin Liu, Rui Zhang, Ge Sun, Bingkun Guo, Fei Du, Shuyan Song, Zhen‐An Qiao, Sheng Dai

**Affiliations:** ^1^ State Key Laboratory of Inorganic Synthesis and Preparative Chemistry Jilin University Changchun Jilin 130012 China; ^2^ Materials Genome Institute Shanghai University Shanghai 200444 China; ^3^ Key Laboratory of Physics and Technology for Advanced Batteries (Ministry of Education) State Key Laboratory of Superhard Materials College of Physics Jilin University Changchun Jilin 130012 China; ^4^ State Key Laboratory of Rare Earth Resource Utilization Changchun Institute of Applied Chemistry Chinese Academy of Sciences Changchun Jilin 130022 China; ^5^ Chemical Sciences Division Oak Ridge National Laboratory Oak Ridge TN 37831 USA

**Keywords:** hierarchically porous structures, macro/mesoporous materials, NASICON‐structured materials, self‐assembly, spinodal decomposition

## Abstract

A general polymer‐assisted spinodal decomposition strategy is used to prepare hierarchically porous sodium super ionic conductor (NASICON)‐structured polyanion‐type materials (e.g., Na_3_V_2_(PO_4_)_3_, Li_3_V_2_(PO_4_)_3_, K_3_V_2_(PO_4_)_3_, Na_4_MnV(PO_4_)_3_, and Na_2_TiV(PO_4_)_3_) in a tetrahydrofuran/ethanol/H_2_O synthesis system. Depending on the boiling point of solvents, the selective evaporation of the solvents induces both macrophase separation via spinodal decomposition and mesophase separation via self‐assembly of inorganic precursors and amphiphilic block copolymers, leading to the formation of hierarchically porous structures. The resulting hierarchically porous Na_3_V_2_(PO_4_)_3_ possessing large specific surface area (≈77 m^2^ g^−1^) and pore volume (≈0.272 cm^3^ g^−1^) shows a high specific capacity of 117.6 mAh g^−1^ at 0.1 C achieving the theoretical value and a long cycling life with 77% capacity retention over 1000 cycles at 5 C. This method presented here can open a facile avenue to synthesize other hierarchically porous polyanion‐type materials.

## Graphical abstract

A general polymer‐assisted spinodal decomposition strategy allows the synthesis of hierarchically porous carbon‐coated sodium super ionic conductor (NASICON)‐structured materials based on multiscale phase separation in both the mesoscopic and macroscopic ranges. The hierarchically porous Na_3_V_2_(PO_4_)_3_ cathode exhibits an excellent activity for sodium‐ion batteries due to the combination of the advantages of the porous framework and inherent NASICON structure of NVP.

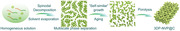

## 1 Introduction

The sodium super ionic conductor (NASICON)‐structured polyanion‐type materials Na*_x_*M_2_(NO_4_)_3_ (N = P^5+^, Mo^6+^, S^6+^, and Si^4+^; M = V, Ti, Zr, Mn, etc.) with 3D large open framework are of significant interest because they allow the reversible and rapid Na^+^ diffusion.^[^ ^1^, ^2^, ^3^ ^]^ In particular, Na_3_V_2_(PO_4_)_3_ (NVP) has been regarded as excellent cathode materials for sodium‐ion batteries (SIBs) due to its high theoretical energy density of 400 Wh kg^−1^ and reversible capacity over 117.6 mAh g^−1^.^[^ ^4^ ^]^ Unfortunately, it is very difficult to realize the theoretical capacity as a result of the poor electronic conductivity of phosphates.^[^ ^5^ ^]^ Furthermore, the large bulk structure of NVP with dense frameworks is bad for transportation of Na^+^ and the uniform deposition of discharge products, thus resulting in the poor performance.^[^ ^6^ ^]^ Up to now, many strategies, primarily including coating NVP active materials with conductive materials to improve the electronic conductivity and downsizing NVP particles to reduce the electron and Na^+^ diffusion paths, have been proposed to address these issues.^[^ ^7^ ^]^ In this case, encapsulation of NVP nanoparticles into 3D porous carbon skeleton has been demonstrated to be an acceptable attempt for increasing the electronic conductivity of NVP.^[^ ^8^, ^9^ ^]^ Although the properties have been improved in some aspects such as rate performance and cycling stability, it also leads to some adverse impacts such as low tap density and volumetric energy density.

Recently, hierarchically porous materials with the combination of periodic mesopores (2–50 nm) and macropores (>50 nm) hold significant potential in energy storage fields owing to their advanced physical and chemical properties as well as superior structural properties such as ultrahigh surface areas, interconnected hierarchical porosity, low density, large accessible space, and micrometer‐scale particle size.^[^ ^10^, ^11^, ^12^, ^13^, ^14^ ^]^ Such characteristics both offer the rapid transportation of electrons and ions and high tap density due to close contact among nanocrystalline particles in the micrometer‐sized hierarchical architecture. In the past few years, the synthesis of hierarchically macro/mesoporous materials is mainly based on the use of surfactants as soft templates to produce mesopores and the preformed colloidal crystals (e.g., polymer beads, silica beads, and foam) as hard templates to create macropores.^[^ ^15^, ^16^, ^17^, ^18^ ^]^ However, this method usually involves laborious and complicated steps, low yields, high cost, and the resulting structural compositions are still limited to a small range, such as metal oxides, silica, and carbon.

Up to now, it is still an enormous challenge to synthesize hierarchically porous NASICON‐structured polyanion‐type materials, for instance NVP, using traditional synthesis methods for porous materials including nanocasting and molecular self‐assembly methods. As an ionic compound, NVP is easy to be dissolved in common solvents during the process of nanocasting, which makes it extremely difficult to maintain the porous structure of product during removal of the hard template process. When using the molecular self‐assembly method, it is really of hardship to regulate the deposition of inorganic precursors to the hydrophilic part of surfactant templates and co‐assemble into porous structure due to the high reactivity of negatively charged phosphate anions (PO_4_^3−^) and positively charged metal ions (V^n+^).^[^ ^19^ ^]^ Moreover, after the high‐temperature treatment (≈700 °C), NVP particles have a strong tendency to sinter into irregular or bulk structure during crystallization process.^[^ ^20^ ^]^ Therefore, the development of a versatile method for the synthesis of porous NVP is highly desirable but remains a great challenge.

**Scheme 1:**
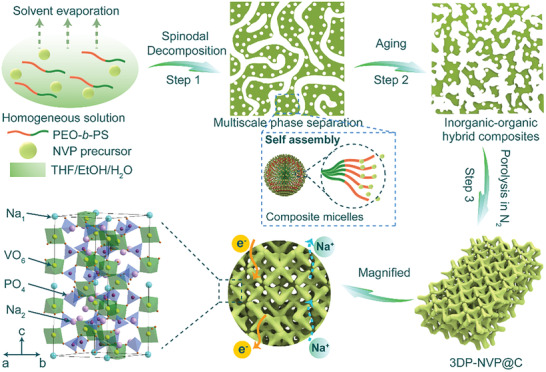
Schematic representation of tentative formation mechanism of 3DP‐NVP@C through the polymer‐assisted spinodal decomposition strategy.

With this aim in mind, we develop a general polymer‐assisted spinodal decomposition strategy for the synthesis of a series of hierarchically porous NASICON‐structured polyanion‐type materials (e.g., Na_3_V_2_(PO_4_)_3_, Li_3_V_2_(PO_4_)_3_, K_3_V_2_(PO_4_)_3_, Na_4_MnV(PO_4_)_3_, and Na_2_TiV(PO_4_)_3_) in a tetrahydrofuran (THF)/ethanol (EtOH)/H_2_O synthesis system (**Scheme** **1**). Depending on their boiling point, the selective evaporation of the solvents induces both mesophase separation via self‐assembly of inorganic precursors and polymers and macrophase separation via spinodal decomposition, resulting in the formation of unique 3D‐interconnected porous structures. The polymers such as amphiphilic poly(ethylene oxide)‐*b*‐polystyrene (PEO‐*b*‐PS) diblock copolymer are used as the structure‐directing agent (SDAs) for mesopores and carbon source to form carbon layer coating active nanocrystals and maintain the stability of porous structure during annealing process. The resulting 3D‐interconnected porous carbon‐coated NVP (abbreviation as 3DP‐NVP@C) nanocomposites possess an open macro/mesoporous structure with large specific surface areas (up to 77 m^2^ g^−1^) and pore volumes (0.272 cm^3^ g^−1^). Owing to their special features, the 3DP‐NVP@C cathode shows a high specific capacity of 117.6 mAh g^−1^ at 0.1 C approaching the theoretical value, a remarkable rate capability of 96.8 mAh g^−1^ at 10 C, and a prominent cyclic stability with 77% capacity retention over 1000 cycles at 5 C.

## 2 Results and Discussion

Figure S1a, Supporting Information, displays the detailed fabrication steps of hierarchically porous NVP. NVP precursor was prepared by adding phosphoric acid and sodium carbonate into the as‐obtained VOC_2_O_4_ aqueous solution. After introducing the SDA of PEO_117_‐*b*‐PS_190_ into NVP precursor (Figure S2, Supporting Information), a transparent blue NVP precursor/PEO_117_‐*b*‐PS_190_/THF/EtOH/H_2_O mixture was obtained. Then, the mixture could slowly evaporate the volatile components at 40 °C (THF and EtOH) and aged at 100^ ^°C to remove solvents completely. Finally, the resulting composites were preheated at 350 °C and pyrolyzed over 700 °C in inert atmosphere, leading to highly crystalline 3DP‐NVP@C. The samples obtained at different calcination temperatures are denoted as 3DP‐NVP@C‐*T*, where *T* refers to the calcination temperatures.

**Figure 1:**
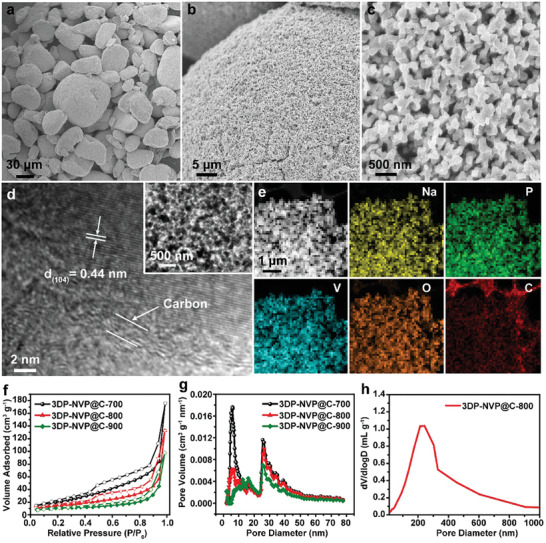
Morphology and microstructure characterization of 3DP‐NVP@C. a–c) SEM images, d) HRTEM image, and e) scanning TEM image and corresponding EDS element mapping of C, Na, O, P, and V for 3DP‐NVP@C‐800. The inset in (d) is TEM image of 3DP‐NVP@C‐800. f) Nitrogen sorption isotherms and g) pore size distribution curves of 3DP‐NVP@C obtained at different calcination temperatures. h) Macropore size distribution determined by mercury intrusion porosimetry of 3DP‐NVP@C‐800.

The morphology and pore structure of 3DP‐NVP@C were characterized by transmission electron microscopy (TEM) and scanning electron microscopy (SEM). SEM images of the products with calcination temperatures ranging from 700–900 °C display hierarchically porous structures that the mesopores and macropores are homogeneously distributed throughout the specimen domain (**Figure** **1**a–c; Figures S3 and S4, Supporting Information). TEM image of 3DP‐NVP@C‐800 shows that the porous skeleton consists of NVP nanocrystals with the diameter of 100–200 nm, mesopores with the diameter of 10–50 nm, and macropores with the diameter of 100–500 nm (the inset in Figure 1d). Distinct lattice fringes with a d‐spacing of 0.44 nm, corresponded to the (104) plane of rhombohedral NVP, can be observed in high‐resolution TEM (HRTEM) image (Figure 1d), which confirms the crystalline pore walls of 3DP‐NVP@C‐800. Moreover, a ≈1.6 nm carbon coating layer with regular lattice fringes on the surface of nanocrystals can also be found, which is greatly beneficial for the electronic conductivity of materials. Scanning TEM (STEM) and energy dispersive spectrometry (EDS) element mapping are employed to study the element distribution of 3DP‐NVP@C‐800 and display the homogeneous distribution of Na, V, P, O, and C (Figure 1e).

The pore structure of 3DP‐NVP@C was investigated by N_2_ adsorption–desorption analysis and mercury intrusion porosimetry. N_2_ sorption isotherms of these samples correspond to characteristic type *I*–*V* curves with a H2‐type hysteresis loop (Figure 1f). The H2‐type hysteresis loop in the pressure (*P/P*_0_) range of 0.4–0.9 indicates the presence of mesopores.^[^ ^21^ ^]^ The uptake at high relative pressure (*P/P*_0_ > 0.9) in each curve confirms that these materials are rich in macropores. The pore size distribution curves for the products, calculated by density functional theory (NLDFT) method, display two types of mesopores of 10 and 30 nm, respectively (Figure 1g). The Brunauer–Emmett–Teller (BET) surface area and pore volume of 3DP‐NVP@C‐700 are as high as 77 m^2^ g^−1^ and 0.272 cm^3^ g^−1^, respectively, (Table S1, Supporting Information). With increasing the calcination temperatures from 800 to 900 °C, the BET surface areas and pore volumes gradually decrease from 51 to 20 m^2^ g^−1^, and from 0.205 to 0.151 cm^3^ g^−1^, respectively. Mercury intrusion porosimetry further reveals that 3DP‐NVP@C‐800 consists of multiple levels of macropore sizes ranging from 100 to 600 nm and the average pore diameter is 270 nm (Figure 1h), which agrees with the observations in TEM and SEM images.

**Figure 2:**
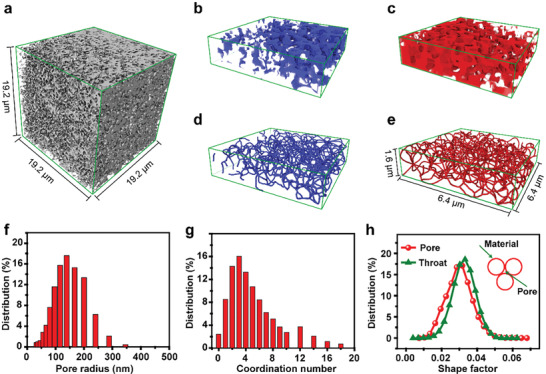
Macrostructure characterization of 3DP‐NVP@C‐800. a) Isosurface visualization of the 3D tomographic reconstruction of macrostructure of 3DP‐NVP@C‐800 obtained from nanoCT data. b–e) Macroporous structure is analyzed with a pore network extraction method based on the maximal ball algorithm, which simplifies the complicated 3D image into skeletal representations. The zoomed images of b) material (blue) and c) porous (red) regions, the skeletal networks for the d) material (blue) regions and the e) porous (red) regions. f) Population distribution of pore radius, g) coordination number, and h) shape factor. Note: the definitions of pore, throat, and shape factor are shown in Figures S7 and S8, Supporting Information.

The nanoscale X‐ray computed tomography (nanoCT) was further employed to characterize the 3D macroporous structure of 3DP‐NVP@C‐800, which cannot destroy the structure of the sample and can reconstruct a relatively large volume of ≈65 µm (Figure S5, Supporting Information). After pretreatment, the 3D‐reconstructed images of 3DP‐NVP@C‐800 show a disordered macrostructure (**Figure** **2**a; Figure S6a and Videos S1 and S2, Supporting Information). To unveil the characteristics of complex macrostructure, we use a pore network extraction method based on the maximal ball algorithm to simplify the 3D images and analyze the composition of macrostructure.^[^ ^22^ ^]^ The ball‐and‐stick model displays that the porous structure of 3DP‐NVP@C‐800 is composed of pore represented by red ball and throat represented by green stick (Figure S6b, Supporting Information). Moreover, the skeletal networks for the material region (blue) and porous region (red) are established by simplifying the corresponding zoomed 3D images, which keep the topological structure and geometrical characteristic of 3DP‐NVP@C‐800 and exhibit a highly interconnected and co‐continuous macrostructure (Figure 2b–e). The skeletal network analyses provide the detailed information of macrostructure, such as the pore size distribution (e.g., pore radius, throat radius, and throat length) and the degree of complexity (coordination number and shape factor). The main population of pore radius for 3DP‐NVP@C‐800 ranges from 50 to 250 nm and the average pore radius is calculated, based on the correction principle of pore and throat (see the Experimental Section, Supporting Information), to be 130 nm (Figure 2f; Table S2, Supporting Information), which is consistent with SEM images and mercury intrusion porosimetry. The throat radius is mainly distributed in the range below 150 nm and the efficient throat length can reach 50–600 nm range (Figure S6c,d, Supporting Information). We also calculate the average coordination number that refers to the number of throats connected to a pore and plays a crucial role in the pore continuity and transport properties.^[^ ^23^ ^]^ The average coordination numbers predicted for the sample is 5, further confirming the well‐interconnected macroporous structure (Figure 2g). We approximate the shape of pores and throats with arbitrary capillaries of cross‐sectional shape due to the complex and highly irregular geometric profiles. The pore shape factor and throat shape factor are widely distributed in the range of 0–0.045, indicating the shapes of pore and throat are cross‐sectional triangle (Figure 2h). The highly interconnected macroporous structure enables the increased interfacial area of electrolyte/electrode and fast diffusion of ions, which is beneficial for the applications in electrochemical systems.

**Figure 3:**
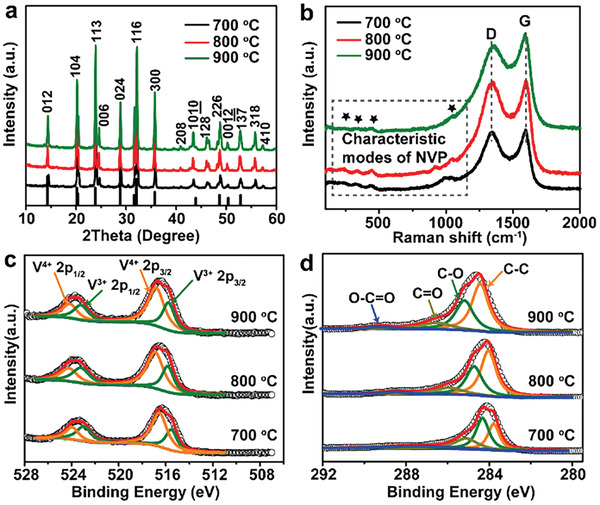
Structural characterizations of 3DP‐NVP@C. a) XRD patterns, b) Raman spectra, c) V 2p, and d) C 1s high‐resolution XPS spectra of 3DP‐NVP@C obtained at different calcination temperatures. The black line in (a) shows the standard patterns of rhombohedral NVP structure (JCPDS no. 53‐0018).

The phase purity and crystallinity of 3DP‐NVP@C were characterized by wide‐angle X‐ray diffraction (WXRD) analysis. All the diffraction peaks can be assigned to rhombohedra phase with *R‐*3*c* space group (JCPDS no. 53‐0018), suggesting the good crystal structure of 3DP‐NVP@C. And increasing the calcination temperatures from 700 to 900 °C improves the crystallinity of the samples (**Figure** **3**a). The structural properties and composition of 3DP‐NVP@C were elucidated by various spectroscopic measurements and thermogravimetric (TG) analysis. The Raman spectra of 3DP‐NVP@C show the characteristic modes of NVP in the range from 50 to 1200 cm^−1^, which can be attributed to the stretching vibrations of PO_4_^3−^. There are two types of carbon bands, including the G (graphite band) bands and D (disorder‐induced phonon mode) situated at 1594 and 1342 cm^−1^, respectively (Figure 3b).^[^ ^24^ ^]^ The intensity ratios of D band to G band (*I*_D_ */I*_G_) for 3DP‐NVP@C‐700, 3DP‐NVP@C‐800, and 3DP‐NVP@C‐900 are 1.01, 0.98, and 0.89, respectively, indicating the relatively higher graphitization degree of 3DP‐NVP@C‐900. X‐ray photoelectron spectroscopy (XPS) reveals the presence of C, Na, O, P, and V signals in all samples (Figure S9, Supporting Information). The existence of two valence states (V^3+^ and V^4+^) of V can be observed in V 2p high‐resolution XPS spectra of 3DP‐NVP@C (Figure 3c). The presence of V^4+^ may be ascribed to the oxidation of the samples in air.^[^ ^25^ ^]^ The C 1s high‐resolution XPS spectra (Figure 3d) of 3DP‐NVP@C can be deconvoluted into four types of carbon, namely, sp^2^‐hybridized carbon (C—C, C=O) and sp^3^‐hybridized carbon (C—O, O—C=O).^[^ ^26^ ^]^ The high content of C—C in 3DP‐NVP@C‐800 and 3DP‐NVP@C‐900 suggests the superior conductivity (Table S3, Supporting Information). Determined from TG analysis and CHN elemental analysis, the 3DP‐NVP@C‐900 possesses minimal carbon content (1.9%) as compared with 3DP‐NVP@C‐700 (4.2%) and 3DP‐NVP@C‐800 (2.7%) (Figure S10 and Table S4, Supporting Information). Due to the low carbon content and large crystalline grains of 3DP‐NVP@C‐900, it possesses the highest tap density of 1.02 g cm^−3^, followed by 3DP‐NVP@C‐800 (0.93 g cm^−3^) and 3DP‐NVP@C‐700 (0.81 g cm^−3^).

**Figure 4:**
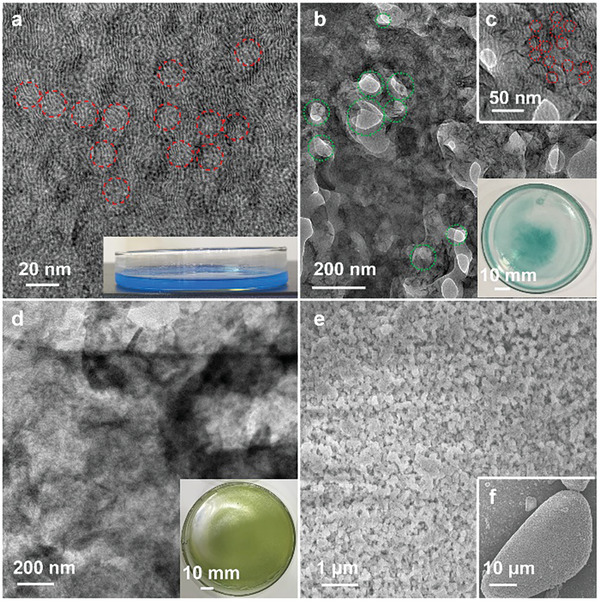
TEM images of a) the NVP precursor/PEO_117_‐*b*‐PS_190_/THF/EtOH/H_2_O mixture after evaporation at 40 °C for 4 h, b,c) the NVP precursor/PEO_117_‐*b*‐PS_190_/THF/EtOH/H_2_O mixture after evaporation at 40 °C for 24 h, and d) the NVP precursor/PEO_117_‐*b*‐PS_190_/THF/EtOH/H_2_O mixture after evaporation at 40 °C for 24 h. e,f) SEM images of the NVP precursor/PEO_117_‐*b*‐PS_190_ mixture after pyrolysis at 350 °C for 5 h. The red dotted circles represent mesopores and the green dotted circles represent macropores. The inset in (a), (b), and (d) is the corresponding photographs.

Based on the above results, we propose a polymer‐assisted spinodal decomposition strategy for the synthesis of 3DP‐NVP@C (Scheme 1). First, the PEO_117_‐*b*‐PS_190_/THF solution is introduced in NVP precursor/H_2_O/EtOH solution to form a transparent blue solution with an obvious Tyndall effect. The dynamic light scattering (DLS) experiments reveal the micellar sizes of 45 nm in the mixture are larger than those of PEO_117_‐*b*‐PS_190_ unimer (≈33 nm) (Figure S11, Supporting Information), indicating the NVP precursor can interact with the PEO block in PEO_117_‐*b*‐PS_190_ by favorable enthalpic interactions (e.g., hydrogen bonding, ionic interactions) and self‐assemble into spherical core–shell composite micelles with the core of PS block and the shell of PEO block/NVP precursor.^[^ ^19^ ^]^ In the initial stage of solvent evaporation at 40 °C for 4 h, these composite micelles can self‐assemble and gradually flock together to form uniform mesostructures driven by the interfacial force of THF and water (**Figure** **4**a; Figure S12, Supporting Information).^[^ ^27^ ^]^ Along with the further evaporation of solvents at 40 °C for 24 h, the removal of highly volatile solvents (THF and EtOH) leads to NVP precursor/PEO_117_‐*b*‐PS_190_ composites more insoluble owing to the strong hydrophobicity of the PS block, and thus the stable mixture enters a supersaturated state‐thermodynamically unstable state. It functions as a driving force to trigger the macrophase separation through spinodal decomposition to form macropores (Figure S13, Supporting Information).^[^ ^28^, ^29^ ^]^ Meanwhile, the composite micelles are gradually destroyed and the NVP precursor grows and gathers, which results in the collapse of pore walls and the combination of mesopores and macropores, thus forming a hierarchically porous structures (step 1, Figure 4b,c; Figure S14, Supporting Information).^[^ ^30^ ^]^ Subsequent removing H_2_O at 100 °C for 24 h further enhances the destruction of mesophases and the growth of NVP precursor, and forms the inorganic–organic hybrid composites (step 2, Figure 4d; Figure S15, Supporting Information). By preheating at 350 °C in N_2_ atmosphere, SEM images indicate the obtained sample possesses a porous structure constituted of small nanoparticles (Figure 4e,f). FT‐IR spectrum of the product reveals the peaks in the range of 1200–2000 cm^−1^, corresponding to the monosubstituted benzene rings in PEO_117_‐*b*‐PS_190_, almost disappear, which confirms the most decomposition of PEO_117_‐*b*‐PS_190_ (Figure S16, Supporting Information). The residual PEO_117_‐*b*‐PS_190_ can be carbonized into rigid carbon to form the carbon coating layer and be in situ used as stabilizer to prevent the uncontrolled aggregation of NVP nanoparticles and maintain the stability of porous structure. After further pyrolysis over 700 °C, NVP precursor can be crystallized without the structure collapse (step 3). Finally, the highly crystalline NVP with hierarchically porous structures is obtained.

The effect of polymers and types of solvents on the porous structure of NVP were also investigated to demonstrate the formation mechanism of 3DP‐NVP@C. In a comparative experiment without PEO_117_‐*b*‐PS_190_, when H_2_O is directly used as the solvent (Figure S1b, Supporting Information), the evaporation of single solvent (H_2_O) cannot be used as the stimulus for macrophase separation, the nonporous bulk NVP (denoted as NPB‐NVP) is obtained (Figure S17a, Supporting Information), suggesting the multicomponent solvents are vital for the macrophase separation. When using EtOH/H_2_O or THF/EtOH/H_2_O as solvents without any polymers (Figure S1c, Supporting Information), the selective evaporation of three solvents at 40 °C for 24 h can act as a trigger to induce macrophase segregation between the H_2_O‐rich phase and the NVP precursor‐rich phase (Figure S18a, Supporting Information). Subsequent thoroughly removing H_2_O at 100 °C for 24 h leaves highly interconnected macropores (Figure S18b,c, Supporting Information). After pyrolysis at 700 °C in N_2_ atmosphere, SEM and TEM images exhibit the porous bulk NVP only possesses the macroporous structures with low pore volumes (Figure S17b–i, Supporting Information), confirming that PEO_117_‐*b*‐PS_190_ is the decisive factor for mesophase segregation.

Under the guidance of the synthesis of 3DP‐NVP@C, the polymer assisted‐spinodal decomposition strategy can be extended to prepare other hierarchically porous NASICON‐structured polyanion‐type materials (e.g., Li_3_V_2_(PO_4_)_3_, K_3_V_2_(PO_4_)_3_, Na_4_MnV(PO_4_)_3_, and Na_2_TiV(PO_4_)_3_) by introduction of corresponding inorganic precursors into the reaction system (Figure S19, Supporting Information). Moreover, the porous polyanion‐type materials can also be synthesized by using other polymers as SDAs, such as triblock copolymer (Pluronic F127 and P123) and poly(ethylene oxide) (PEO‐5000), and so forth, and the corresponding results are shown in Figure S20 and Table S5, Supporting Information. These extending results suggest that our proposed strategy can be generalized for synthesizing hierarchically porous polyanion‐type materials of other topologies by rationally selecting the polymers and inorganic precursors.

**Figure 5:**
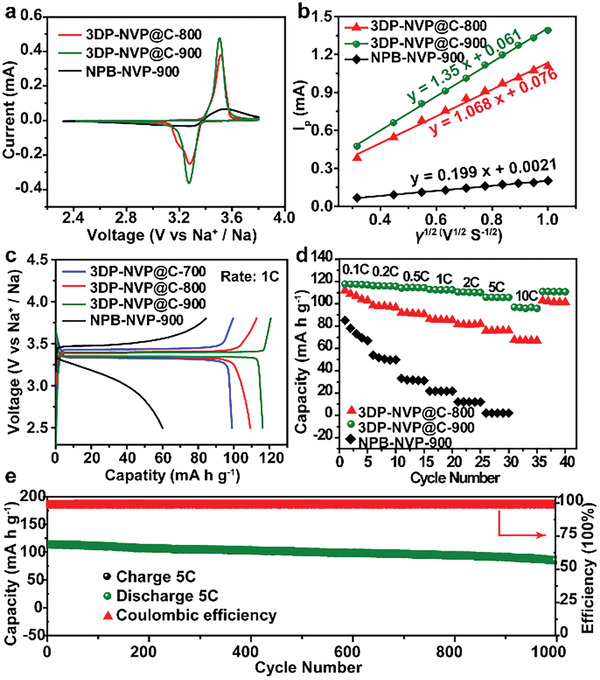
Electrochemical performance of 3DP‐NVP@C and NPB‐NVP‐900 cathodes. a) Cyclic voltammetry measured at 0.1 mV s^−1^ of 3DP‐NVP@C‐800, 3DP‐NVP@C‐900, and NPB‐NVP‐900. b) Linear fitting of *I*_p_ versus *v* ^1/2^ curves of 3DP‐NVP@C‐800, 3DP‐NVP@C‐900, and NPB‐NVP‐900 from the redox peaks in CV curves. c) The initial charge–discharge curves of 3DP‐NVP@C‐700, 3DP‐NVP@C‐800, 3DP‐NVP@C‐900, and NPB‐NVP‐900. d) Rate performances of 3DP‐NVP@C‐800, 3DP‐NVP@C‐900, and NPB‐NVP‐900. e) Long‐term cycle life of 3DP‐NVP@C‐900 for 1000 cycles at 5 C.

The electrochemical properties of 3DP‐NVP@C obtained at different calcination temperatures and NPB‐NVP‐900 (the sample calcined at 900 °C in N_2_ atmosphere) were evaluated by the assembly of half‐cells with metallic Na as anode. Cyclic voltammetry (CV) measured at different scan rates were performed to investigate the electrochemical behaviors. A pair of well‐defined redox peaks in the range of 2.3–3.8 V are observed in CV curves measured at 0.1 mV s^−1^ for the samples, which can be attributed to the V^4+^/V^3+^ redox couple reaction accompanied by Na^+^ insertion/extraction from the NVP lattice matrix (**Figure** **5**a).^[^ ^31^ ^]^ Compared with the other samples, the current of 3DP‐NVP@C‐900 highest, and the reduction and oxidation peaks of 3DP‐NVP@C‐900 shift toward higher and lower potentials, respectively, suggesting its low polarization, which may be ascribed to the high crystallinity, improved conductivity, and porous framework of 3DP‐NVP@C‐900. The well‐defined redox peaks are still maintained even at a high scan rate of 1 mV s^−1^, indicating the excellent rate performance of 3DP‐NVP@C‐900 (Figure S21, Supporting Information). The well linear relationship between *v* ^1/2^ (the square root of scan rate) and *I*_p_ (the peak current density) confirms the diffusion‐controlled reaction during the sodium storage in NVP electrodes (Figure 5b). According to Randles–Sevcik equation,^[^ ^32^ ^]^ the sodium‐ion diffusion coefficient (*D*) of 3DP‐NVP@C‐900 is calculated to be 4.07 × 10^−11^ cm^2^ s^−1^, higher than those of 3DP‐NVP@C‐800 (2.51 × 10^−11^ cm^2^ s^−1^) and NPB‐NVP‐900 (8.87 × 10^−13^ cm^2^ s^−1^), which indicates the fast charge transfer kinetics and improved rate capability of 3DP‐NVP@C‐900.

The galvanostatic discharge/charge measurements of the products were carried out at the dis‐/charge rate of 1 C (1 C = 117 mA g^−1^) over a potential range of 2.3–3.8 V (vs Na^+^/Na). The distinct charge and discharge plateaus at around 3.4 V, assignable to the redox pair of V^3+^/V^4+^,^[^ ^33^ ^]^ is observed for 3DP‐NVP@C (Figure 5c), which is consistent with the CV profiles. Among these samples, the designed 3DP‐NVP@C‐900 shows a smallest voltage gap between charge and discharge plateaus and decreased potential slope before the cutoff voltages, suggesting the lowest polarization. As expected, the 3DP‐NVP@C‐900 exhibits the highest capacity of 116.4 mAh g^−1^, followed by 3DP‐NVP@C‐800 (110.1 mAh g^−1^), 3DP‐NVP@C‐700 (99.2 mAh g^−1^), and then NPB‐NVP‐900 (60.1 mAh g^−1^) (Figure 5c). After 100 cycles, the capacity retention of 3DP‐NVP@C‐900 remains 95%, which is much higher than those of 3DP‐NVP@C‐800 (80%) and NPB‐NVP‐900 (40%) (Figure S22, Supporting Information). The superior performance of 3DP‐NVP@C‐900, compared with 3DP‐NVP@C‐700 and 3DP‐NVP@C‐800, can be ascribed to the improved conductivity and crystallinity. Moreover, the hierarchically porous structure is beneficial for the increased interfacial area of electrolyte/electrode and guarantees the fast transportation of electrons.^[^ ^34^ ^]^ Thus, the performance of 3DP‐NVP@C‐900 is much higher than that of NPB‐NVP‐900.

The rate performances of the samples were also studied and shown in Figure 5d. The introduction of porous structure and conductive carbon coating into the bulk NVP greatly enhances the rate performance. The 3DP‐NVP@C‐900 electrode delivers an improved discharge capacity of 117.6 mAh g^−1^ at the initial rate of 0.1 C, achieving the theoretical value. With increasing the current rate to 0.2, 0.5, 1.0, and 2.0 C, 5 C, and, 10 C, the discharge capacities are still as high as 116.5, 115.3, 113.4, 111.2, 106.6, and 96.8 mAh g^−1^, respectively. When the current density is turned back to 0.1 C, the specific capacity of 115.2 mAh g^−1^ is recovered again for the 3DP‐NVP@C‐900, confirming the remarkable reversibility. Furthermore, the 3DP‐NVP@C‐900 also exhibits the lowest polarization voltage as compared with 3DP‐NVP@C‐800 and NPB‐NVP‐900 with increasing the current rates (Figure S23, Supporting Information). The impedance spectra (EIS) measurements were further employed to elucidate the reason for the improved performance of 3DP‐NVP@C‐900. The impedance arc radius for 3DP‐NVP@C‐900 is much smaller than that of 3DP‐NVP@C‐800, confirming the high crystallinity is beneficial for the charge transfer kinetics. The introduction of hierarchically porous structure greatly decreases the charge transfer resistance of bulk NVP, demonstrating that the 3D‐interconnected channels can offer more efficient electron/ion transport (Figure S24, Supporting Information).^[^ ^35^, ^36^ ^]^ Benefiting from these merits, the 3DP‐NVP@C‐900 shows an excellent cycling stability that 77% of the initial capacity is retained after 1000 cycles at high current rate of 5 C, which is much higher than those of other samples (Figure 5e; Figure S25, Supporting Information). The Coulombic efficiency of 3DP‐NVP@C‐900 is close to 100% during cycling, indicating that the Na^+^ insertion/extraction mechanism is highly reversible.

## 3 Conclusion

In summary, we develop a facile and general polymer‐assisted spinodal decomposition strategy to synthesize a series of hierarchically porous NASICON‐structured materials. Based on the boiling point of solvents, the controlled solvent evaporation induces the multiscale phase separation in both the mesoscopic and macroscopic ranges. During this process, the polymers such as PEO_117_‐*b*‐PS_190_ are used as SDAs and carbon sources to form carbon‐coated active nanocrystals and prevent the collapse of porous structure during the crystallization of inorganic precursors. So, this method can be generalized for synthesizing other hierarchically porous polyanion‐type materials with defined porosity and interconnectivity by rationally selecting the polymers and inorganic precursors. Combining the advantages of the porous framework and inherent NASICON structure of NVP, the obtained 3DP‐NVP@C electrodes exhibit the excellent sodium storage performances. The method may open new horizons in constructing advanced hierarchically porous materials, such as NaFePO_4_, NaTi_2_(PO_4_)_3_, NaV_1‐_ *_x_*M*_x_*PO_4_F, Na_3_(VO_1‐_ *_x_*PO_4_)_2_F_1+_ *_2x_* (0 < *x* < 1), and so on.

## Conflict of Interest

The authors declare no conflict of interest.

## Supporting information

Supporting Information

Supplemental Video 1

Supplemental Video 2

## Data Availability

Research data are not shared.

## References

[1] D. Wang , X. Bie , Q. Fu , D. Dixon , N. Bramnik , Y.‐S. Hu , F. Fauth , Y. Wei , H. Ehrenberg , G. Chen , F. Du , Nat. Commun. 2017, 8, 15888. 28660877 10.1038/ncomms15888 PMC5493763

[2] Z. Jian , Y.‐S. Hu , X. Ji , W. Chen , Adv. Mater. 2017, 29, 1601925. 10.1002/adma.201601925 28220967

[3] Y. Qi , Z. Tong , J. Zhao , L. Ma , T. Wu , H. Liu , C. Yang , J. Lu , Y.‐S. Hu , Joule 2018, 2, 2348.

[4] Q. Zheng , H. Yi , X. Li , H. Zhang , J. Energy Chem. 2018, 27, 1597.

[5] Q. An , F. Xiong , Q. Wei , J. Sheng , L. He , D. Ma , Y. Yao , L. Ma , Adv. Energy Mater. 2015, 5, 1401963.

[6] X. Cao , A. Pan , B. Yin , G. Fang , Y. Wang , X. Kong , T. Zhu , J. Zhou , G. Cao , S. Liang , Nano Energy 2019, 60, 312.

[7] W. Ren , X. Yao , C. Niu , Z. Zheng , K. Zhao , Q. An , Q. Wei , M. Yan , L. Zhang , L. Mai , Nano Energy 2016, 28, 216.

[8] Y. Jiang , Z. Yang , W. Li , L. Zeng , F. Pan , M. Wang , X. Wei , G. Hu , L. Gu , Y. Yu , Adv. Energy Mater. 2015, 5, 1402104.

[9] Y. Fang , L. Xiao , X. Ai , Y. Cao , H. Yang , Adv. Mater. 2015, 27, 5895. 26305519 10.1002/adma.201502018

[10] L. Chen , M. Sun , Z. Wang , W. Yang , Z. Xie , B.‐L. Su , Chem. Rev. 2020, 120, 11194. 32915551 10.1021/acs.chemrev.0c00016

[11] L. Wu , Y. Li , Z. Fu , B.‐L. Su , Natl. Sci. Rev. 2020, 7, 1667. 10.1093/nsr/nwaa183 PMC8288509 34691502

[12] J. Jin , L. Wu , S. Huang , M. Yan , H. Wang , L. Chen , T. Hasan , Y. Li , B.‐L. Su , Small Methods 2018, 2, 1800171.

[13] Y. Boyjoo , H. Shi , Q. Tian , S. Liu , J. Liang , Z. Wu , M. Jaroniec , J. Liu , Energy Environ. Sci. 2021. 10.1039/D0EE03316B.

[14] S. Yu , G. Xing , L. Chen , T. Ben , B.‐L. Su , Adv. Mater. 2020, 32, 2003270. 10.1002/adma.202003270 32930443

[15] R. Zhang , D. Shen , M. Xu , D. Feng , W. Li , G. Zheng , R. Che , A. Elzatahry , D. Zhao , Adv. Energy Mater. 2014, 4, 1301725.

[16] Y. Liu , K. Lan , A. Bagabas , P. Zhang , W. Gao , J. Wang , Z. Sun , J. Fan , Small 2016, 12, 860. 26708310 10.1002/smll.201503420

[17] T. Zhao , Y. Ren , J. Yang , L. Wang , W. Jiang , A. Elzatahry , A. Alghamdi , Y. Deng , D. Zhao , W. Luo , J. Mater. Chem. A 2016, 4, 16446.

[18] T. Sun , N. Shan , L. Xu , J. Wang , J. Chen , A. A. Zakhidov , R. H. Baughman , Chem. Mater. 2018, 30, 1617.

[19] B. Bastakoti , Y. Li , S. Guragain , M. Pramanik , S. Alshehri , T. Ahamad , Z. Liu , Y. Yamauchi , Chem. ‐ Eur. J. 2016, 22, 7463. 27087399 10.1002/chem.201600435

[20] D. Yang , Z. Lu , X. Rui , X. Huang , H. Li , J. Zhu , W. Zhang , Y. Lam , H. Hng , H. Zhang , Q. Yan , Angew. Chem. Int. Ed. 2014, 53, 9352. 10.1002/anie.201404615 24990356

[21] H. Xiong , L. Wu , Y. Liu , T. Gao , K. Li , Y. Long , R. Zhang , L. Zhang , Z. Qiao , Q. Huo , X. Ge , S. Song , H. Zhang , Adv. Energy Mater. 2019, 9, 1901634.

[22] H. Dong , M. Blunt , Phys. Rev. E 2009, 80, 036307. 10.1103/PhysRevE.80.036307 19905212

[23] C. Jo , J. Hwang , W. Lim , J. Lim , K. Hur , J. Lee , Adv. Mater. 2018, 30, 1703829. 10.1002/adma.201703829 29271508

[24] Y. Cai , X. Cao , Z. Luo , G. Fang , F. Liu , J. Zhou , A. Pan , S. Liang , Adv. Sci. 2018, 5, 1800680. 10.1002/advs.201800680 PMC6145241 30250805

[25] J. Guo , P. Wang , X. Wu , X. Zhang , Q. Yan , H. Chen , J. Zhang , Y. Guo , Adv. Mater. 2017, 29, 1701968. 10.1002/adma.201701968 28639347

[26] E. Wang , M. Chen , X. Liu , Y. Liu , H. Guo , Z. Wu , W. Xiang , B. Zhong , X. Guo , S. Chou , S. Dou , Small Methods 2018, 3, 1800169.

[27] W. Luo , Y. Li , J. Dong , J. Wei , J. Xu , Y. Deng , D. Zhao , Angew. Chem. Int. Ed. 2013, 52, 10505. 10.1002/anie.201303353 23943495

[28] J. Hwang , C. Jo , K. Hur , J. Lim , S. Kim , J. Lee , J. Am. Chem. Soc. 2014, 136, 16066. 25338137 10.1021/ja5091172

[29] W. Lim , C. Jo , A. Cho , J. Hwang , S. Kim , J. Han , J. Lee , Adv. Mater. 2019, 31, 1806547. 10.1002/adma.201806547 30484914

[30] T. Inoue , Prog. Polym. Sci. 1995, 20, 119.

[31] S. Li , Y. Dong , L. Xu , X. Xu , L. He , L. Mai , Adv. Mater. 2014, 26, 3545. 24633680 10.1002/adma.201305522

[32] Y. Xu , Q. Wei , C. Xu , Q. Li , Q. An , P. Zhang , J. Sheng , L. Zhou , L. Mai , Adv. Energy Mater. 2016, 6, 1600389.

[33] J. Fang , S. Wang , Z. Li , H. Chen , L. Xia , L. Dinga , H. Wang , J. Mater. Chem. A 2016, 4, 1180.

[34] T. Wei , G. Yang , C. Wang , Nano Energy 2017, 39, 363.

[35] H. Xiong , H. Zhou , G. Sun , Z. Liu , L. Zhang , L. Zhang , F. Du , Z. Qiao , S. Da , Angew. Chem., Int. Ed. 2020, 59, 11053. 10.1002/anie.202002051 32173989

[36] P. Wang , S. Sun , Y. Jiang , Q. Cai , Y. Zhang , L. Zhou , S. Fang , J. Liu , Y. Yu , ACS Nano 2020, 14, 15577. 33108161 10.1021/acsnano.0c06250

