# Supplementary material for: A Polymer‐Assisted Spinodal Decomposition Strategy toward Interconnected Porous Sodium Super Ionic Conductor‐Structured Polyanion‐Type Materials and Their Application as a High‐Power Sodium‐Ion Battery Cathode
Source: Adv Sci (Weinh). 2021 Mar 20;8(11):2004943. doi: 10.1002/advs.202004943 (PMC8188202; doi:10.1002/advs.202004943)
Supplement: Supplementary file 1 — Supporting Information [file ADVS-8-2004943-s002.pdf]

WILEY-VCH

© 2021 Wiley-VCH GmbH

ADVANCED SCIENCE
Open Access

Supporting Information

for *Adv. Sci.*, DOI: 10.1002/advs.202004943

A Polymer-Assisted Spinodal Decomposition Strategy toward Interconnected Porous Sodium Super Ionic Conductor-Structured Polyanion-Type Materials and their Application as High-Power Sodium-Ion Battery Cathode

Hailong Xiong, Ruicheng Qian, Zhilin Liu, Rui Zhang, Ge Sun, Bingkong Guo, Fei Du, Shuyan Song, Zhen-An Qiao, and Sheng Dai*

WILEY-VCH

© 2021 Wiley-VCH GmbH

Supporting Information

A Polymer-Assisted Spinodal Decomposition Strategy toward Interconnected Porous Sodium Super Ionic Conductor-Structured Polyanion-Type Materials and their Application as High-Power Sodium-Ion Battery Cathode

Hailong Xiong, Ruicheng Qian, Zhilin Liu, Rui Zhang, Ge Sun, Bingkong Guo, Fei Du, Shuyan Song, Zhen-An Qiao, and Sheng Dai*

Dr. H. Xiong, Z. Liu, R. Zhang, Prof. Z.-A. Qiao
State Key Laboratory of Inorganic Synthesis and Preparative Chemistry, Jilin University, Changchun, Jilin 130012 (China)
E-mail: qiaozhenan@jlu.edu.cn

R. Qian, Prof. B. Guo
Materials Genome Institute, Shanghai University, Shanghai, 200444 (China)

G. Sun, Prof. F. Du
Key Laboratory of Physics and Technology for Advanced Batteries (Ministry of Education), State Key Laboratory of Superhard Materials, College of Physics, Jilin University, Jilin 130012 (China)

Prof. S. Song
State Key Laboratory of Rare Earth Resource Utilization, Changchun Institute of Applied Chemistry, Chinese Academy of Sciences, Changchun 130022 (China)

Prof. S. Dai
Chemical Sciences Division, Oak Ridge National Laboratory, Oak Ridge, TN, 37831, USA

Keywords: hierarchically porous structure, macro/mesoporous materials, spinodal decomposition, self-assembly, NASICON-structured materials

Experimental Procedures

Main Text Paragraph.

Materials: Vanadic oxide, oxalic acid dehydrate, lithium carbonate, sodium carbonate, potassium carbonate, manganese acetate tetrahydrate, tetrabutyl titanate, phosphoric acid, copper (I) bromide, styrene, pyridine, and Al_2O_3 were purchased from Aladdin. Pluronic F127, P123, monomethoxy poly(ethylene oxide) (PEO-5000), N,N,N',N'',N''-Pentamethyldiethylenetriamine, 2-bromoisobutyryl bromide were purchased from Sigma-Aldrich. Tetrahydrofuran (THF) and anhydrous ethylether (EtOH) were of analytical grade and obtained from Sinopharm Chemical Reagent Co. (Shanghai, China). The electrolyte, acetylene black, PVDF, and NMP were purchased from Aladdin. The coin cells, Na metal, and Al foil were purchased from Shenzhen Biyuan Electronics Co., Ltd.

Synthesis of PEO_{117}-b-PS_{190} block copolymers: The amphiphilic PEO_{117}-*b*-PS_{190} diblock copolymers were prepared by atom-transfer radical polymerization (ATRP) as follows.[S1] Firstly, to obtain a macroinitiator PEO-Br, monomethoxy PEO-5000 (20.0 g) was dissolved in THF (120 mL) and pyridine (20 mL) was then added. The homogeneous solution was placed in an ice-water bath, and 6.0 g of 2-bromoisobutyrylbromide was added dropwise under continuous magnetic stirring. After stirring at 30 ^{o}C for 24 h, 100 mL of ether was added, and the white PEO-Br was precipitated from the solution. After collection by filtration, it was dried in a vacuum at 30 ^{o}C. Secondly, 5.0 g of PEO-Br was added to a boiling flask together with 0.15 g of CuBr, 0.5 g of N,N,N',N'',N''-Pentamethyldiethylenetriamine, and 35 mL of styrene. The flask was degassed and immersed in an oil bath at 110 ^{o}C. After reaction for 3 h with stirring, the system was cooled down to room temperature and the product was dissolved in 100 mL of THF and filtered with Al_2O_3 to remove the catalyst. The PEO_{117}-*b*-PS_{190} diblock copolymer was precipitated from the filtrate by adding 200 mL of ether, and then dried at 50 ^{o}C in a vacuum for 6 h. The degree of polymerization and molecular weight of PEO_{117}-*b*-

PS_{190} were characterized by gel permeation chromatography (GPC). According to GPC, the diblock copolymer PEO_{117}-*b*-PS_{190} possesses narrow molecular-weight distribution (M_n = ~24760) with polydispersity index (PDI) of 1.06 (Figure S2).

Synthesis of 3D-interconnected porous carbon-coated NASICON-structured polyanion-type materials: In a typical synthesis of 3D-interconnected porous carbon-coated $Na_3V_2(PO_4)_3$ (denoted as 3DP-NVP@C), 0.5 mmol V_2O_5 and 2 mmol $H_2C_2O_4$ $2H_2O$ were dissolved into 20 mL deionized water and vigorously stirred at 80 °C for 1 h to obtain a transparent blue VOC_2O_4 aqueous solution. Then 1.5 mmol H_3PO_4, 0.75 mmol Na_2CO_3, and 10 mL EtOH were added into the solution. After stirring for 1 h, the transparent blue solution was naturally cooled to room temperature. The obtained solution was added into the polymeric solution containing 0.2 g PEO_{117}-*b*-PS_{190} and 10 mL THF. Along with further stirring for 2 h, the resultant solution was cast onto 90 mm petri dish and placed on a hot-plate at 40 °C inside a fume hood to obtain a transparent blue membrane without any cracks. Subsequent aging at 100 ^{o}C for 24 h in an oven, a green uniform membrane was obtained. In the end, 3DP-NVP@C-700 were obtained from the precursor by preheating the membrane at 350 °C for 5 h followed by annealing at 700 ^{o}C for 8 h in nitrogen atmosphere with a heating rate of 5 °C min^{-1} in a single-temperature zone tube furnace (OTF-1200X). A series of experiments with different annealing temperatures and polymers were carried out in order to identify the optimal synthesis recipe. The detailed experimental procedures and phenomena are shown in Figure S1a.

The synthesis of other NASICON-structured polyanion-type materials were completed identically to that of 3DP-NVP@C by using the same amount of precursors instead of V_2O_5 as the starting materials.

Synthesis of nonporous bulk NVP (denoted as NPB-NVP-700): 0.5 mmol V_2O_5 and 2 mmol $H_2C_2O_4$ $2H_2O$ were dissolved into 20 mL deionized water and vigorously stirred at 80 °C for 1 h to obtain a transparent blue VOC_2O_4 aqueous solution. Then 1.5 mmol H_3PO_4 and 0.75

mmol Na_2CO_3 were added into the solution. After stirring for 1 h, the transparent blue solution was naturally cooled to room temperaturethe. The resultant solution was cast onto 90 mm petri dish and placed on a hot-plate at 40 °C inside a fume hood. After evaporation of solvents. The obtained NVP precursor was placed into an oven and aged at 100 ^oC for 24 h to completely remove the solvents. Then, the as-synthesized NVP precursor was calcined at 350 °C for 5 h followed by annealing at 700 ^oC for 8 h (ramp rate 5 °C min^{-1}) in nitrogen atmosphere in a single-temperature zone tube furnace (OTF-1200X) to crystallize the NVP precursor, resulting in the highly crystalline NPB-NVP-700. A series of experiments with different annealing temperatures were carried out in order to identify the optimal synthesis recipe. The detailed experimental procedures and phenomena are shown in Figure S1b.

Synthesis of porous bulk NVP*:* 0.5 mmol V_2O_5 and 2 mmol $H_2C_2O_4$ $2H_2O$ were dissolved into 20 mL deionized water and vigorously stirred at 80 °C for 1 h to obtain a transparent blue VOC_2O_4 aqueous solution. Then 1.5 mmol H_3PO_4, 0.75 mmol Na_2CO_3, 10 mL EtOH, and 10 mL THF were added into the solution. After stirring for 1 h, the transparent blue solution was naturally cooled to room temperaturethe. The resultant solution was cast onto 90 mm petri dish and placed on a hot-plate at 40 °C inside a fume hood. After evaporation of solvents. The obtained NVP precursor was placed into an oven and aged at 100 ^oC for 24 h to completely remove the solvents. Then, the as-synthesized NVP precursor was calcined at 350 °C for 5 h followed by annealing at 700 ^oC for 8 h (ramp rate 5 °C min^{-1}) in nitrogen atmosphere in a single-temperature zone tube furnace (OTF-1200X) to crystallize the NVP precursor, resulting in the highly crystalline porous bulk NVP. The detailed experimental procedures and phenomena are shown in Figure S1c.

Electrochemical characterization: The electrochemical properties were characterized by assembling CR2032 type coin cells in a glove box filled with pure argon gas. The electrolyte used in the present study consisted of 1 M $NaClO_4$ in propylene carbonate (PC) and ethylene

carbonate (EC) (1/1, v/v) with 2% fluoroethylene carbonate (FEC). The amount of electrolyte is about 130 μL. Cathode electrodes were obtained by coating a slurry mixed of active materials (70 wt%), aetylene black (20 wt%), and polyvinylidene fluoride (PVDF) (10 wt%) in N-methyl-2-pyrrolidone (NMP) onto an aluminum foil and dried in a vacuum oven at 100 °C overnight. The mass loading of active material is about 0.9 mg cm^{-2}. Galvanostatic charge/discharge cycling behavior was investigated in a potential range of 2.5-3.8 V versus Na^+/Na with a multichannel battery testing system (LAND CT2001A). Electrochemical impedance spectroscopy (EIS) was characterized in a frequency range of 100 K to 0.01 Hz using an electrochemical working station (Solartron analytical 1470 Cell Test System) under open circuit potential conditions. And the voltage amplitude of the sine perturbation signal was 5 mV. Cyclic voltammetry (CV) tests were performed from 2.5-3.8 V vs. Na/Na^+ at relevant scan rates using a Solartron analytical 1470 Cell Test System.

The Na^+ diffusion coefficients (*D*) can be calculated based on the CV curves using the Randles-Sevcik equation:

$$I_p = 2.69 \times 10^5 n^{3/2} A D^{1/2} C v^{1/2} \qquad \text{(Equation S1)}$$

Where I_p, n, A, C, and $v^{1/2}$ represent the peak current density (mA), the number of electrons per molecule, the active surface area of the electrode (cm^2), the concentration of Na^+ in the electrode (mol cm^{-3}), and the square root of scan rate, respectively.

Characterizations: X-ray powder diffraction (XRD) patterns were measured using a Bruker D8 Advance X-ray diffractometer with monochromatic Cu Kα irradiation. N_2 sorption isotherms were measured at 77 K with a Quantachrome Nova 4200e. Mercury intrusion porosimetry was measured on a Micro Active Auto Pore V 9600. X-ray photoelectron spectroscopy (XPS) measurements were measured on an ESCALab220i-XL electron spectrometer. Transmission electron microscopy (TEM) analysis was performed on FEI Tenia G2 F20 microscope operated at 200 kV. Scanning Electron Microscopy (SEM) analysis was conducted on JEOL JSM-6700F field-emission scanning electron microscope operated under

15 kV. The weight loss of the samples was measured using a thermogravimetric analyzer (TGA Q50) in air with a heating rate of 10 °C min^{-1}. Raman spectra were performed on a Renishaw *via* Raman microscope. Gel-permeation chromatography (GPC) was recorded on an Agilent 1260 GPC system with monodispersed polystyrene as standards. The particle sizes were analyzed by dynamic light scattering (DLS) technique taken on a Malvern Zetasizer (Malvern Instruments, Southborough, US). Detection was carried out in a backscattering mode (scattering angle 173 ^{o}). Viscosity of the solutions was set at 1.20 cP, temperature at 25 ^{o}C.

Characterizations of macrostructure: Nano CT data were obtained at the MicroXRM L200 nano-CT scanner. A Fresnel zone plate lens was used to magnify the X-ray images to achieve a resolution of up to 65 μm resolution. The X-ray source voltage of nano-CT was constant at 40 kV and the photon energy of the X-ray was 8 keV. A series of 2D X-ray images (for a total of 360 images) were collected while the sample was rotated by 180°. These image sets were reconstructed by a computed tomography technique to produce the 3D images. We use the image processing software (Avizo Amira) to enhance the contrast of 3D digital display and remove the noise to distinguish the porous structure and material.

Definition of pore and throat size: The maximum sphere algorithm is used to extract the pore network model of macrostructure, as shown in Figure S7, subscript *p* and *t* represent the pore and throat, respectively.[S2, S3]

$$l_t = l_{ij} - l_i - l_j \qquad \text{(Equation S2)}$$

Where l_t stands for the throat length, l_{ij} represents the total throat length (Euclidean distance between the central points of pore *i* and pore *j*), l_i and l_j are the lengths of pore *i* and pore *j*, respectively.

$$l_i = l_i^t(1 - 0.6\frac{r_t}{r_i}) \qquad \text{(Equation S3)}$$

$$l_j = l_j^t(1 - 0.6\frac{r_t}{r_j}) \qquad \text{(Equation S4)}$$

Where r_i, r_j and r_t are the inner diameters of pores *i* and *j* and throat, respectively. l_i^t and l_j^t are the distances from the centers of pores *i* and *j* of to the center of the throat.

Definition of shape factor: We approximate the shape of pores and throats with arbitrary capillaries of cross-sectional shape due to they have complex and highly irregular geometric profiles. The irregularity of cross-section is expressed by a dimensionless shape factor *G*.

$$G = \frac{VL}{A_s^2} \approx \frac{A}{P^2} \qquad \text{(Equation S5)}$$

Where *V, L, A_s, A, and P* represent volume, length, surface area, cross sectional area, and perimeter of pore or throat space, respectively. The shape factors of different cross-sectional shapes are different. As shown in Figure S8, the shape factor of a circle (1/4π) is larger that those of square (1/16) and triangle (0~$\sqrt{3}$/36).

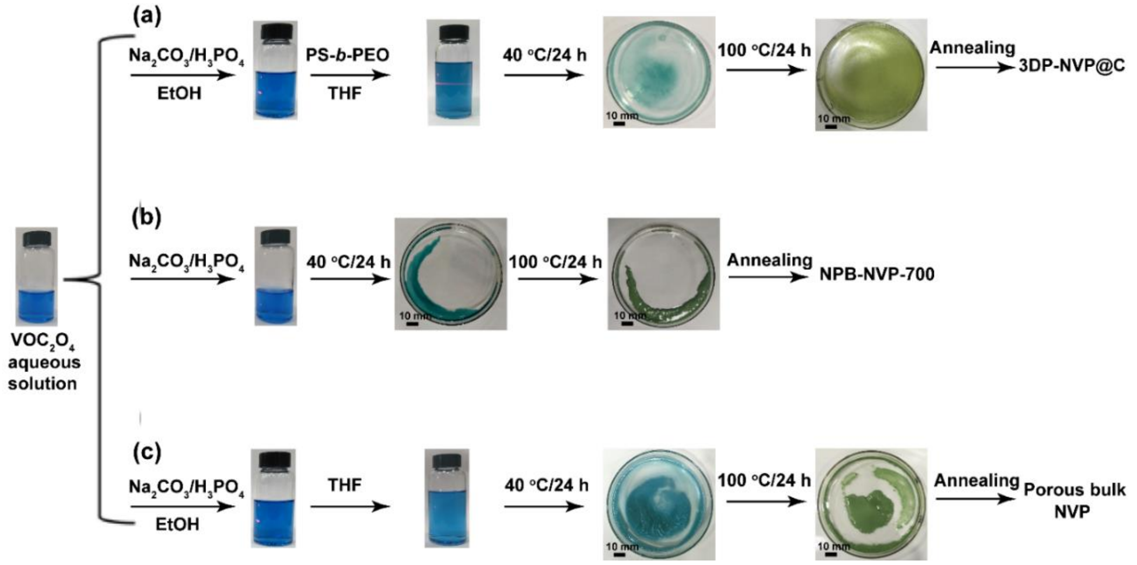

Figure S1. Related photographs of the synthesis processes for (a) 3DP-NVP@C, (b) NPB-NVP, and (c) porous bulk NVP, respectively.

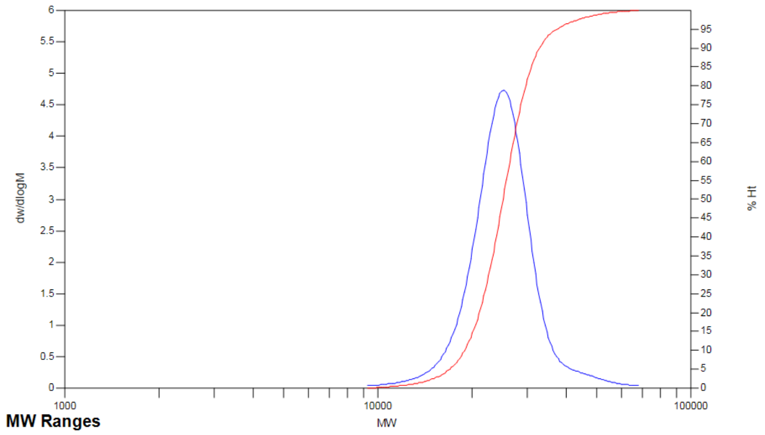

Figure S2. Gel permeation chromatography (GPC) traces of PEO_{117}-*b*-PS_{190}, which show narrow molecular-weight distributions with polydispersity index (PDI) of 1.06.

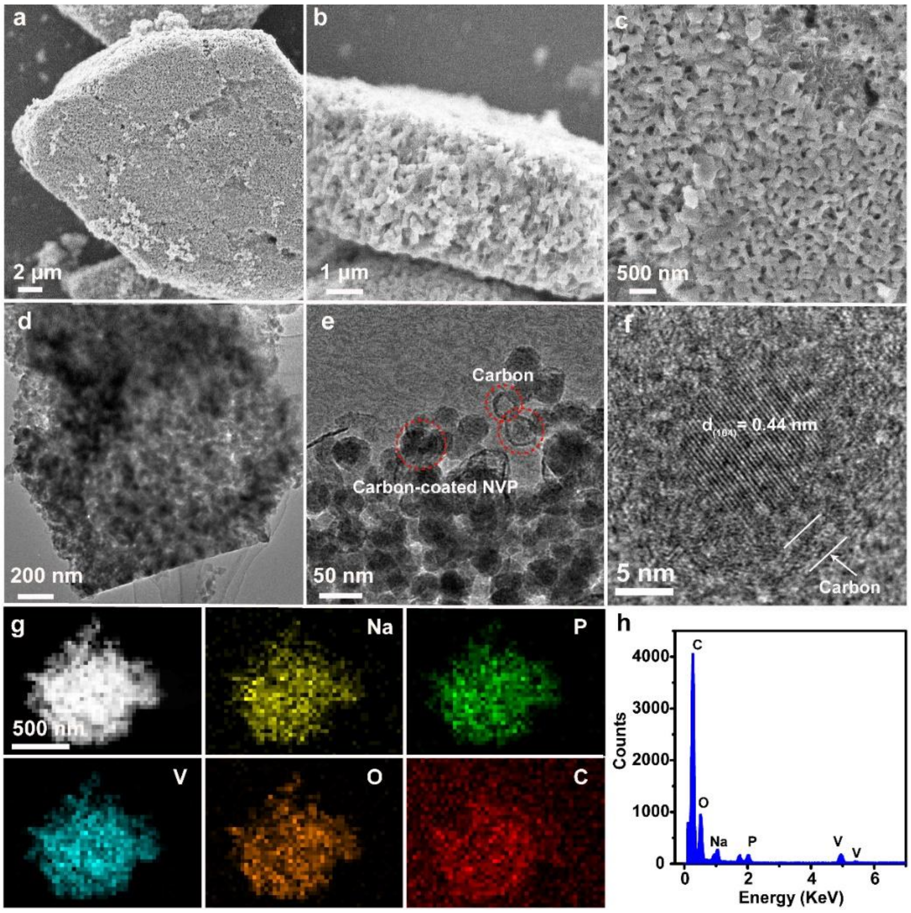

Figure S3. (a-c) SEM images, (d, e) TEM image, (f) HRTEM, (g) scanning TEM image and corresponding EDS element mapping of C, Na, O, P, and V, and (h) EDX spectrum of 3DP-NVP@C-700.

SEM images taken along both the surface and cross-section of the sample clearly indicate that the obtained 3DP-NVP@C-700 is composed of densely packed carbon-coated nanocrystals and possess a large-domain porous structure (Figure S3a-c). TEM images further confirm the porous skeleton consisted of carbon-coated nanocrystals (Figure S3d and e). HRTEM image of 3DP-NVP@C-700 clearly reveals that several layers of carbon shell are coated on the surface of NVP nanocrystals with the thickness of a few nanometers, demonstrating the successful formation of a nano core–shell structure (Figure S3f).

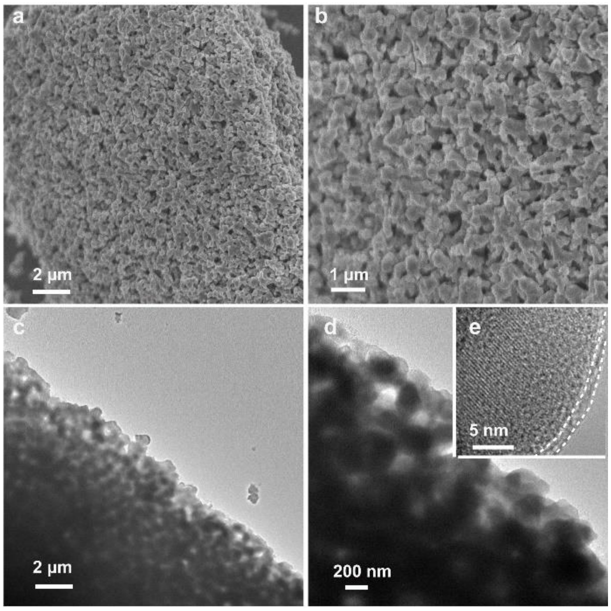

Figure S4. (a, b) SEM images, (c, d) TEM images, and (e) HRTEM image of 3DP-NVP@C-900.

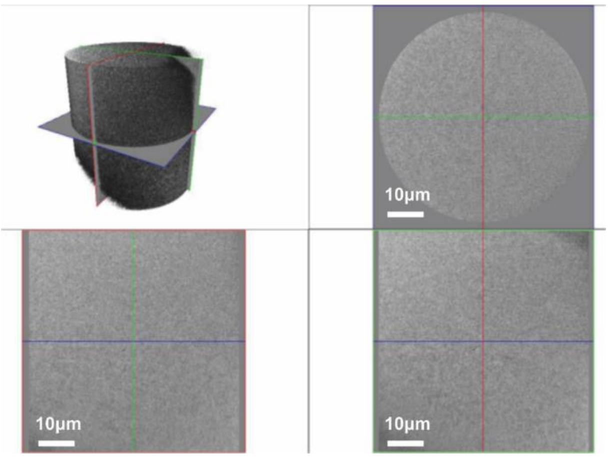

Figure S5. Three-dimensional digital display of 3DP-NVP@C-800 obtained from nanoCT data at a large volume of ~65 µm.

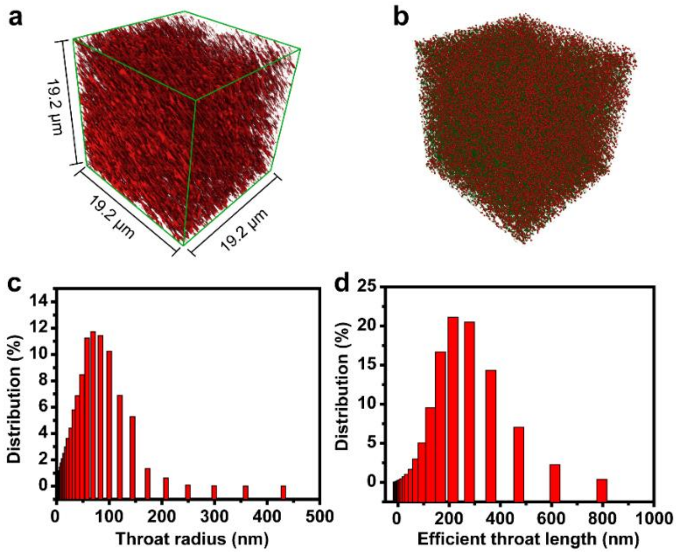

Figure S6. (a) The skeletal networks for porous region and (b) ball-and-stick model of 3DP-NVP@C-800 obtained from nanoCT data. Quantitative structural analysis of skeletal networks for porous region: population distribution of (c) throat radius and (d) efficient throat length.

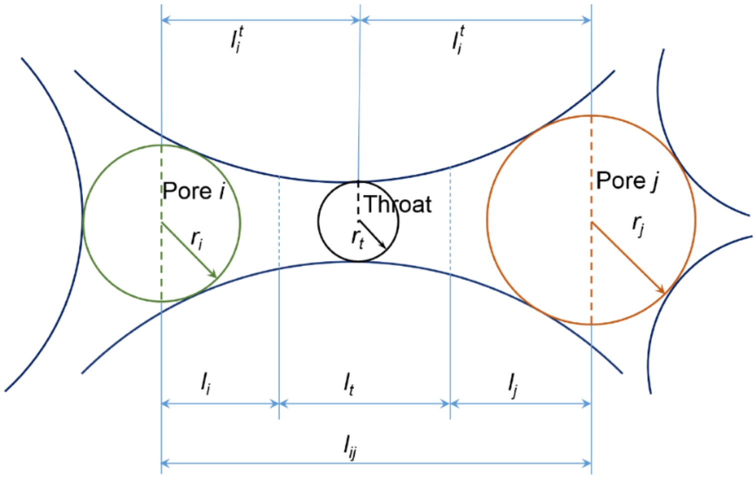

Figure S7. Schematic representations of the length correction for pores and throats. The l_t stands for the throat length, l_{ij} represents the total throat length (Euclidean distance between the central points of pore *i* and pore *j*), l_i and l_j are the lengths of pore *i* and pore *j*, respectively. The r_i, r_j and r_t are the inner diameters of pores *i* and *j* and throat, respectively. The l_i^t and l_j^t are the distances from the centers of pores *i* and *j* of to the center of the throat, respectively.

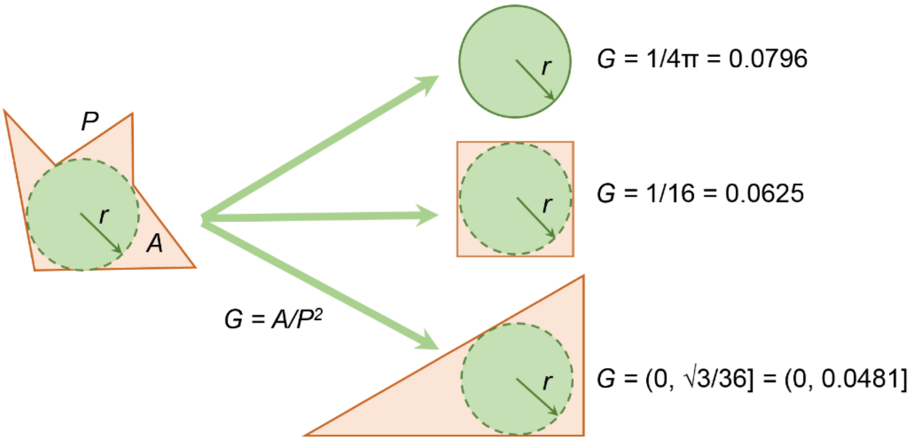

Figure S8. The dimensionless shape factor of pore network elements. The G, V, L, A_s, A, *and* P represent shape factor, volume, length, surface area, cross sectional area, and perimeter of pore or throat space, respectively.

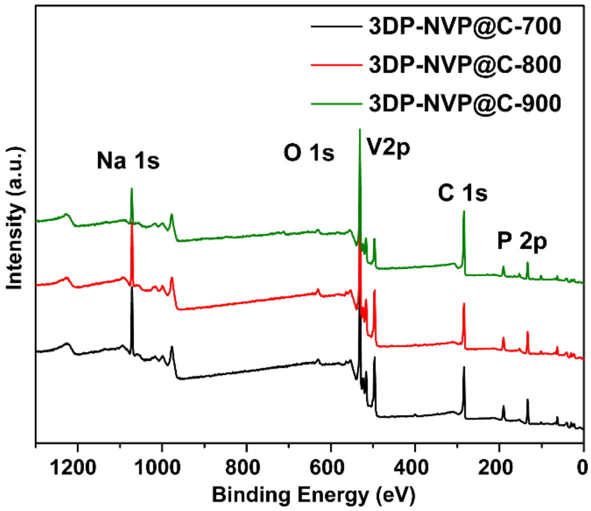

Figure S9. XPS spectra of 3DP-NVP@C obtained at different calcination temperatures.

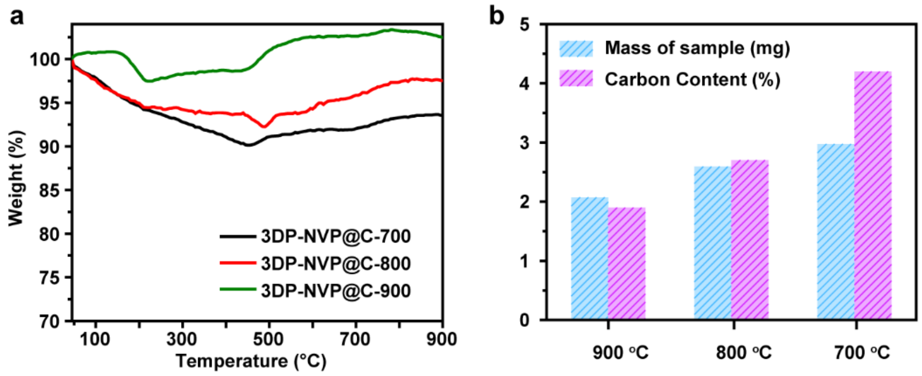

Figure S10. (a) TG curves and (b) CHN elemental analysis of 3DP-NVP@C obtained at different calcination temperatures. The increase of TGA curves of the samples after 300 °C is due to the oxidation of V^{3+} in the NVP to V^{4+} and V^{5+}. The carbon content of the samples is determined by CHN elemental analysis, the corresponding results are shown in Table S4.

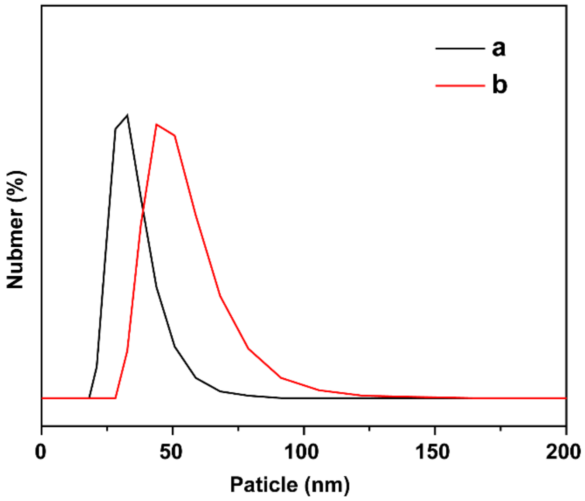

Figure S11. DLS particles size distributions of (a) PEO_{117}-*b*-PS_{190}/THF/EtOH/H_2O mixture and (b) NVP precursor/PEO_{117}-*b*-PS_{190}/THF/EtOH/H_2O mixture.

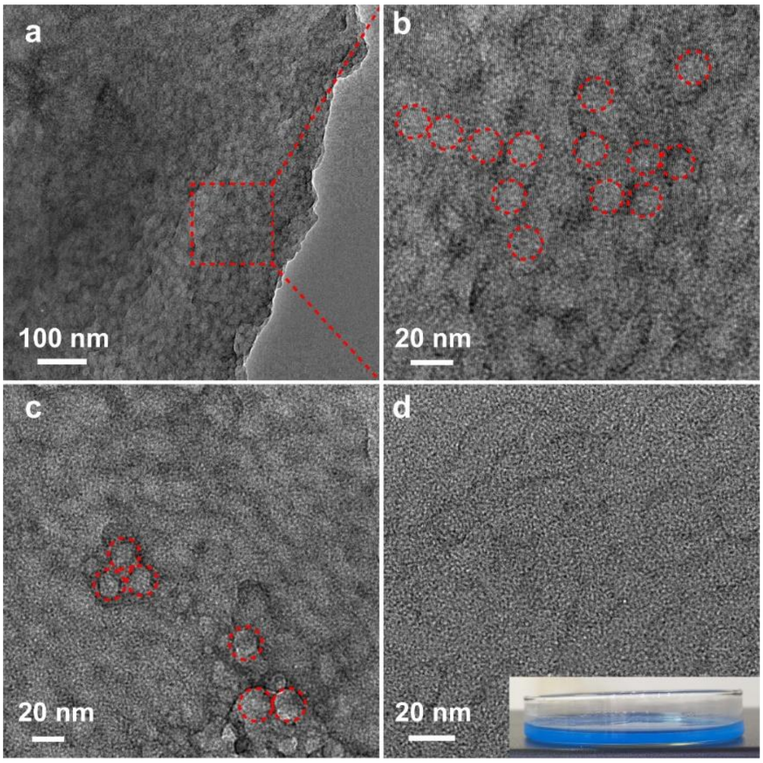

Figure S12. (a-d) TEM images of the NVP precursor/PEO_{117}-*b*-PS_{190}/THF/EtOH/H_2O mixture after evaporation at 40 ^oC for 4 h. The inset in (d) is the corresponding photograph.

After evaporation of solvents for 4 h, the whole reaction system is still a homogeneous mixture (the inset in Figure S12d), confirming the strong interaction between NVP precursor and PEO_{117}-*b*-PS_{190}. TEM images of the sample show a uniform mesostructure (the red circles represent the mesopores), indicating the NVP precursor and PEO_{117}-*b*-PS_{190} can self-assemble into mesophases (Figure S12a-d).

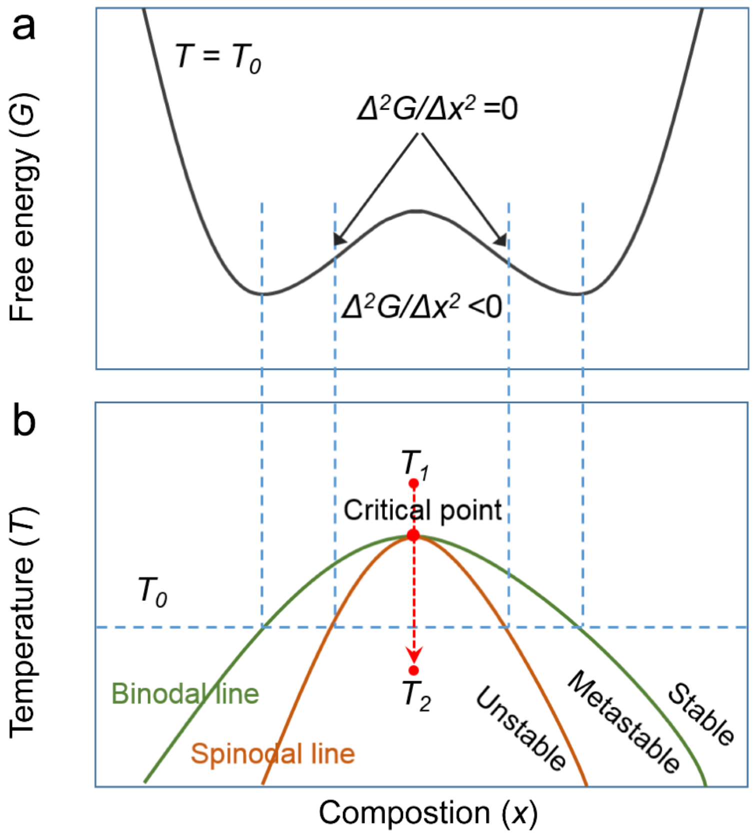

Figure S13. Schematic representations of (a) Gibbs free energy curve of mixture and (b) phase diagram for a ternary system at a specific temperature (T = T_o).

Spinodal decomposition of polymer mixture is a well-known physical phenomenon in polymer science. Driven by the external physical or chemical stimuli, such as elevated temperature or solvent evaporation, the stable multicomponent mixture enters a thermodynamically unstable state and is thus separated into two phases in a short time.[S4-S6] For a miscible system, a stable and homogeneous solution prepared at temperature T_1 can enter a thermodynamically untastable state by increasing the temperature from T_1 to T_2. When the components are located under the spinodal line, spinodal decomposition occurs.

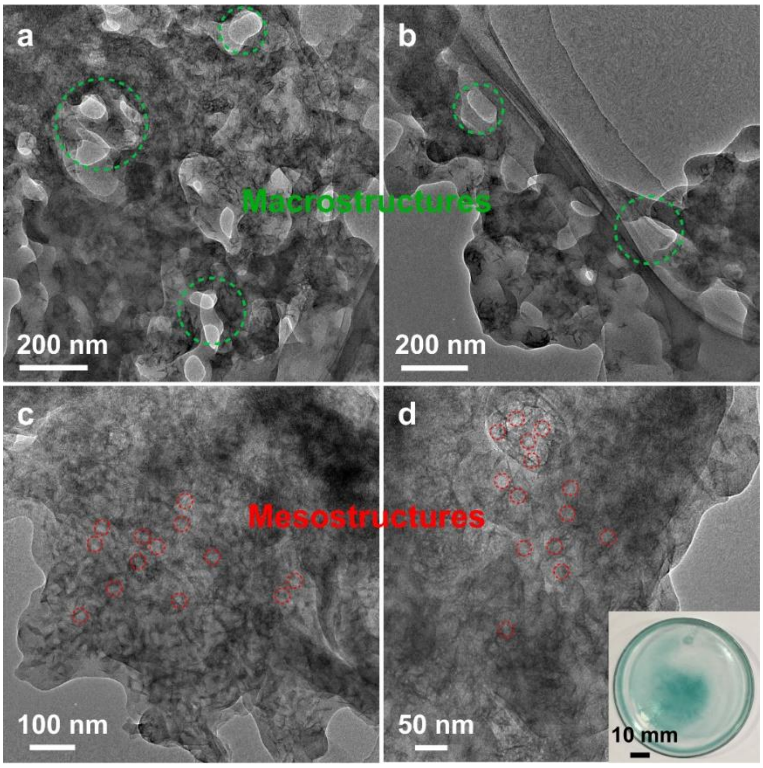

Figure S14. (a-d) TEM images of the NVP precursor/PEO_{117}-*b*-PS_{190}/THF/EtOH/H_2O mixture after evaporation at 40 ^{o}C for 24 h. The inset in (d) is the corresponding photograph. The red circles represent the mesopores and the green circles represent the macropores.

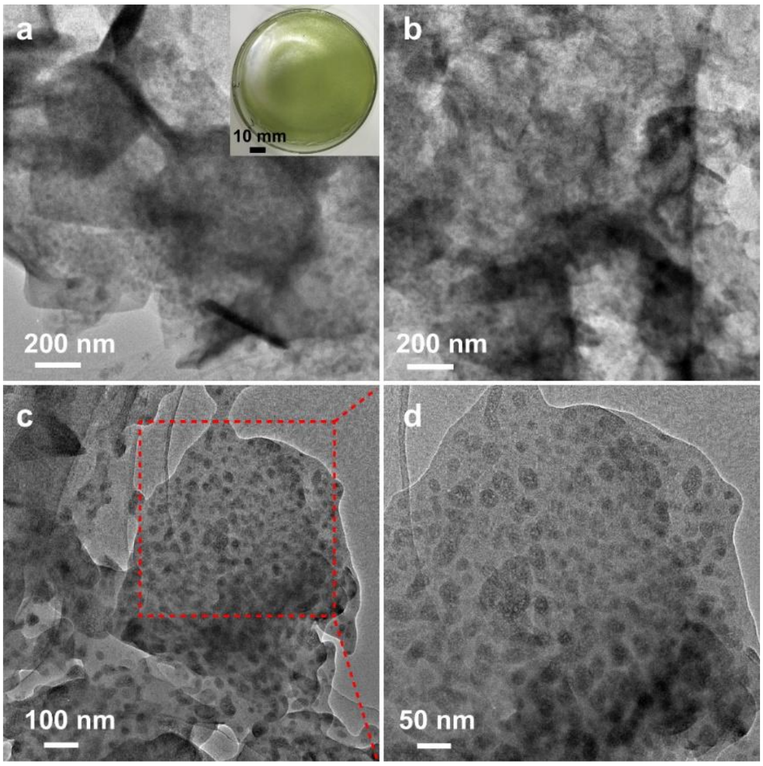

Figure S15. (a-d) TEM images of the NVP precursor/PEO_{117}-*b*-PS_{190}/THF/EtOH/H_2O mixture after evaporation at 100 ^oC for 24 h. The inset in (a) is the corresponding photograph.

After completely removing the solvents at 100 ^oC, a green uniform membrane without any cracks is obtained, confirming the strong interaction between NVP precursor and PEO_{117}-*b*-PS_{190} (the inset in Figure S15a).

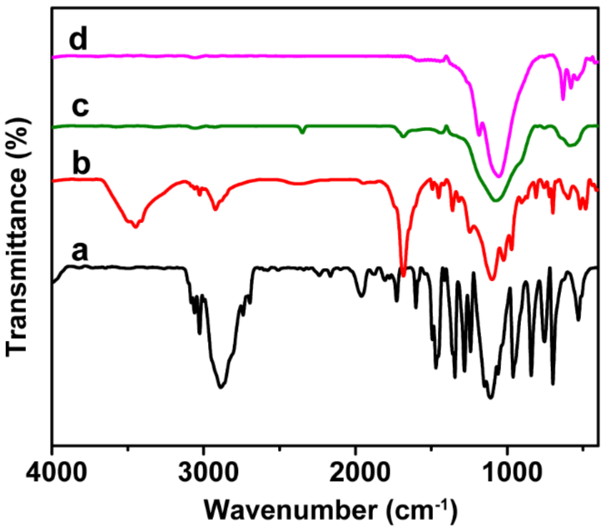

Figure S16. FT-IR spectra of (a) PEO_{117}-*b*-PS_{190}, (b) the NVP precursor/PEO_{117}-*b*-PS_{190} mixture after evaporation at 100 ^{o}C, (c) the NVP precursor/PEO_{117}-*b*-PS_{190} mixture after calcination at 350 ^{o}C, and (d) 3DP-NVP@C-700.

The FT-IR spectra of PEO_{117}-*b*-PS_{190} and NVP precursor/PEO_{117}-*b*-PS_{190} mixture after evaporation at 100 ^{o}C display five peaks appearing at 1200–2000 cm^{-1}, which can be attributed to the monosubstituted benzene rings (Figure S16a and b).[S1] After calcination at 350 ^{o}C, the peaks in the range of 1200–2000 cm^{-1} are almost disappeared, confirming the decomposition of PEO_{117}-*b*-PS_{190} (Figure S16c). FT-IR spectrum of 3DP-NVP@C-700 shows the characteristic absorptions at 1187 and 625 cm^{-1} are assigned to the asymmetric stretching vibrations of PO_4 units and the stretching vibrations of V^{3}–O^{2-} bonds in VO_6 octahedra, respectively (Figure S16d). The peaks at 1058 and 544 cm^{-1} are attributed to the bending vibrations of the P–O bonds of PO_4 tetrahedra.[S7]

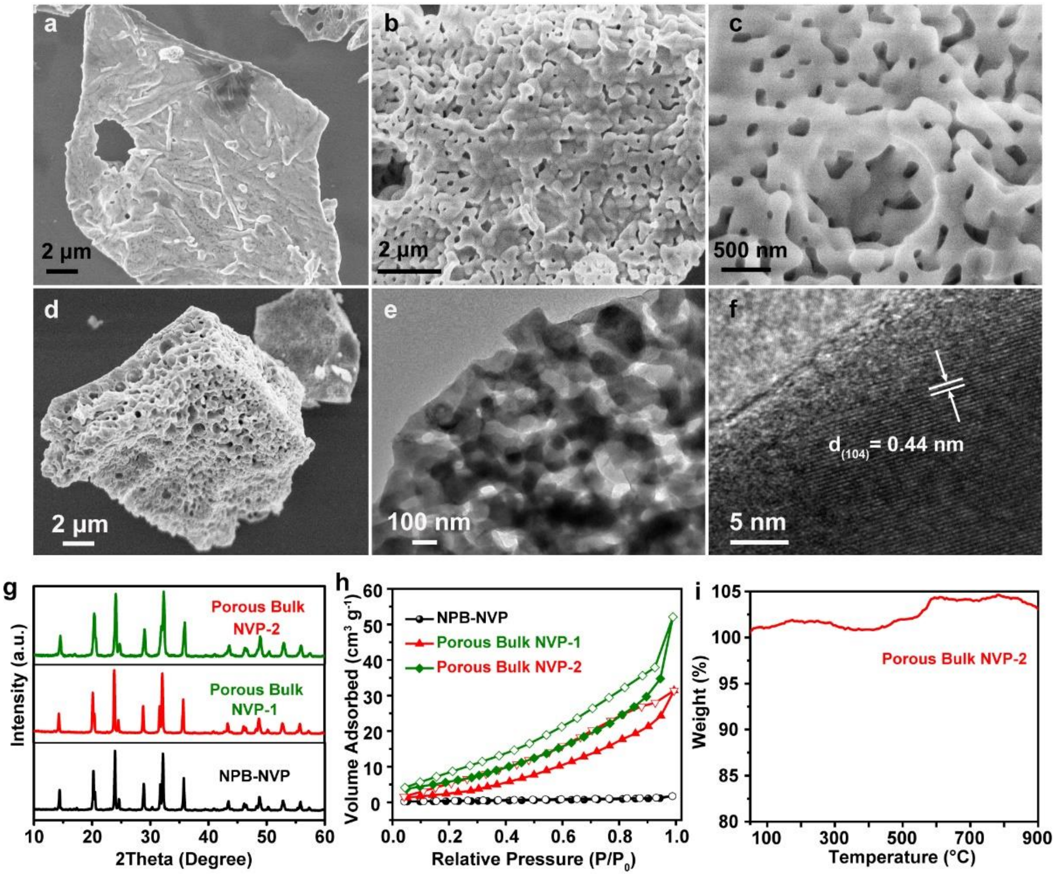

Figure S17. (a) SEM image of NPB-NVP synthesized by evaporation of a NVP precursor/H_2O solution in an open dish of diameter 90 mm placed in a fume hood, followed by calcining at 700 ^oC in inert atmosphere. (b, c) SEM images of porous bulk NVP-1 synthesized by evaporation of a NVP precursor/EtOH/H_2O solution in an open dish of diameter 90 mm placed in a fume hood, followed by calcining at 700 ^oC in inert atmosphere. (d) SEM image, (e) TEM image, (f) HRTEM image, and (i) TG curve of porous bulk NVP-2 synthesized by evaporation of a NVP precursor/THF/EtOH/H_2O solution in an open dish of diameter 90 mm placed in a fume hood, followed by calcining at 700 ^oC in inert atmosphere. (g) XRD patterns and (h) nitrogen sorption isotherms of NBP-NVP, porous bulk NVP-1, and porous bulk NVP-2.

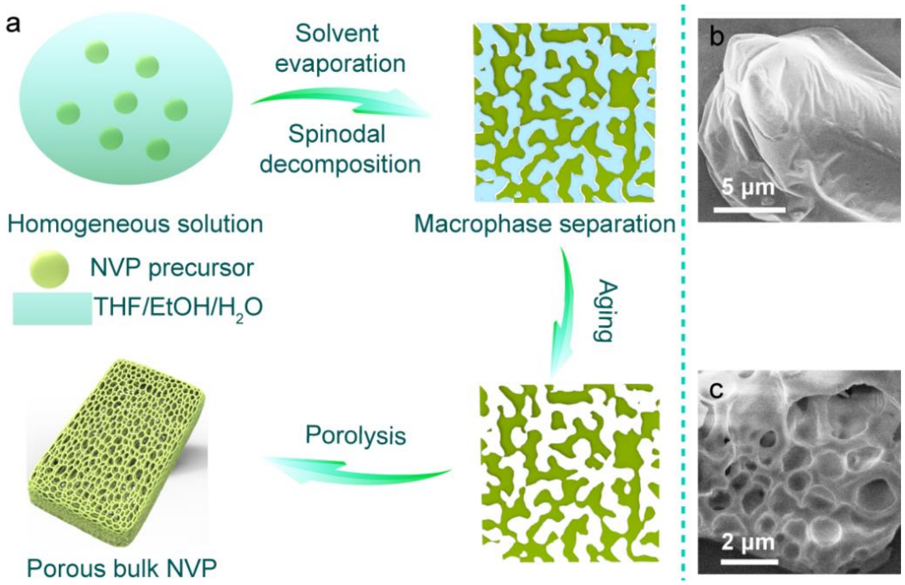

Figure S18. (a) Schematic representation of macrophase separation for the synthesis of 3D porous bulk NVP. SEM images of (b) NVP precursor/THF/EtOH/H_2O mixture after evaporation at 40 ^{o}C and (c) NVP precursor/THF/EtOH/H_2O mixture after thermal curing at 100 °C.

When we use EtOH/H_2O or THF/EtOH/H_2O as solvents without PEO_{117}-*b*-PS_{190}, the preferential evaporation of volatile components (THF or EtOH) at 40 ^{o}C for 24 h can act as a trigger to induce macrophase segregation between the H_2O-rich phase and the NVP precursor-rich phase (Figure S18b), which means that PEO_{117}-*b*-PS_{190} is the decisive factor for mesophase segregation. Subsequent thoroughly removing H_2O at 100 ^{o}C for 24 h with the prolonged evaporation process leaves co-continuous macropores (Figure S18c). After calcination in inert atmosphere, the porous bulk NVP are obtained.

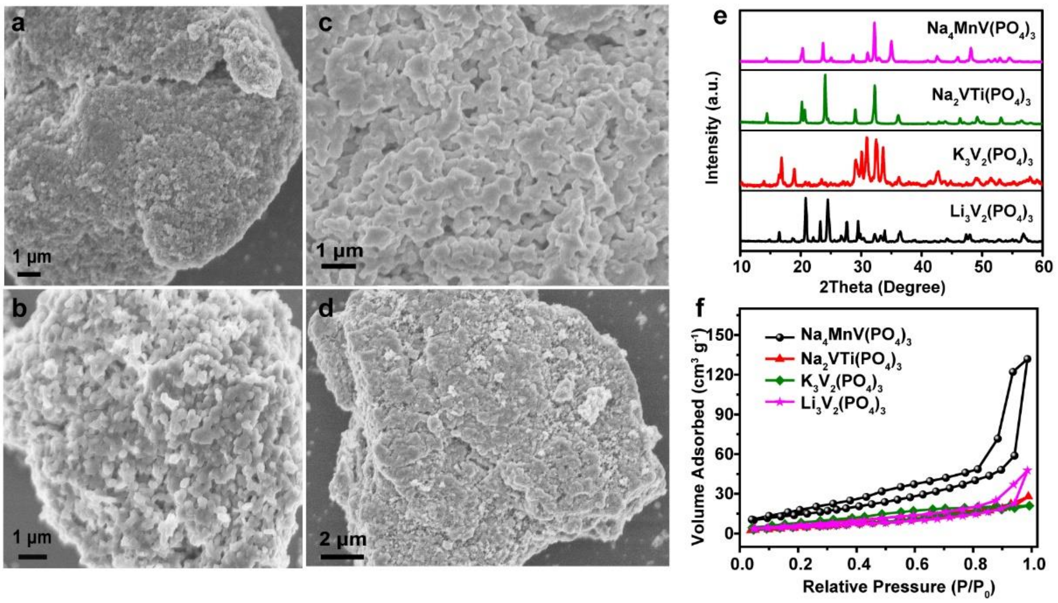

Figure S19. SEM images of hierarchically porous (a) $Li_3V_2(PO_4)_3$, (b) $K_3V_2(PO_4)_3$, (c) $Na_2TiV(PO_4)_3$ and (d) $Na_4MnV(PO_4)_3$. (e) XRD patterns and (f) nitrogen sorption isotherms of hierarchically porous $Li_3V_2(PO_4)_3$, $K_3V_2(PO_4)_3$, $Na_2TiV(PO_4)_3$, and $Na_4MnV(PO_4)_3$.

The polymer assisted-spinodal decomposition strategy can be extended to prepare other hierarchically porous NASICON-structured polyanion-type materials (e.g., $Li_3V_2(PO_4)_3$, $K_3V_2(PO_4)_3$, $Na_4MnV(PO_4)_3$, and $Na_2TiV(PO_4)_3$) by introduction of corresponding inorganic precursors into the reaction system. According to SEM images, all of polyanion-type materials possess hierarchically porous structures and the frameworks of materials consist of nanocrystals (Figure S19a-d). XRD patterns of the samples show all diffraction peaks match well with their corresponding standard data, confirming their crystalline nature (Figure S19e). The N_2 sorption isotherms confirm these materials have moderate surface areas and pore volumes (Figure S19f). The BET surface areas of all of materials are calculated to be 53 m^2 g^{-1} for $Li_3V_2(PO_4)_3$, 19 m^2 g^{-1} for $K_3V_2(PO_4)_3$, 18 m^2 g^{-1} for $Na_4MnV(PO_4)_3$, and 20 m^2 g^{-1} for $Na_2TiV(PO_4)_3$ (Table S5).

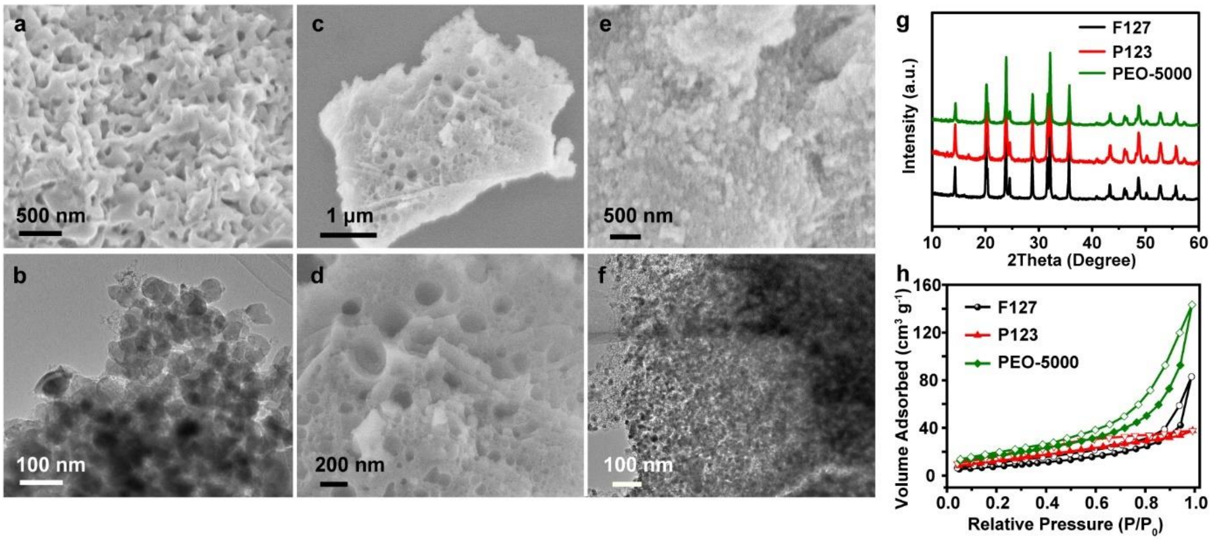

Figure S20. (a) SEM image and (b) TEM image of $Na_3V_2(PO_4)_3$ using F127 as a SDA instead of PEO_{117}-*b*-PS_{190}. (c, d) SEM images of $Na_3V_2(PO_4)_3$ using P123 as a SDA instead of PEO_{117}-*b*-PS_{190}. (e) SEM image and (f) TEM image of $Na_3V_2(PO_4)_3$ using PEO-5000 as a SDA instead of PEO_{117}-*b*-PS_{190}. (g) XRD patterns and (h) nitrogen sorption isotherms of $Na_3V_2(PO_4)_3$ using different polymers as SDAs instead of PEO_{117}-*b*-PS_{190}.

The porous $Na_3V_2(PO_4)_3$ can also be synthesized by using other polymers as SDAs, such as triblock copolymer (Pluronic F127 and P123) and poly(ethylene oxide) (PEO-5000), and so forth. The corresponding results are shown in Figures S20 and the detailed N_2 sorption data are summarized in Table S5.

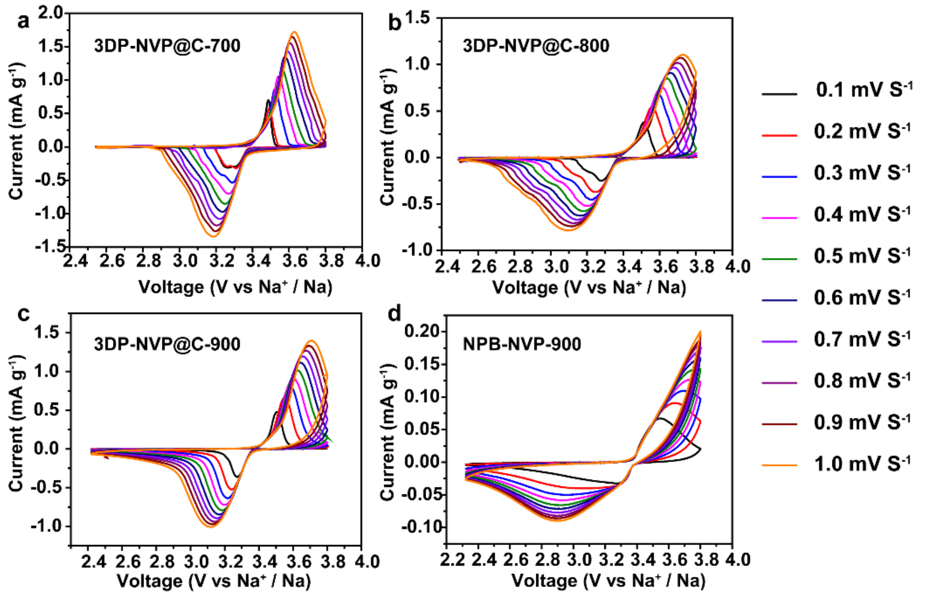

Figure S21. Cyclic voltammetry of (a) 3DP-NVP@C-700, (b) 3DP-NVP@C-800, (c) 3DP-NVP@C-900, and (d) NPB-NVP-900 at different scan rates from 0.1 to 1.0 mV S^{-1}.

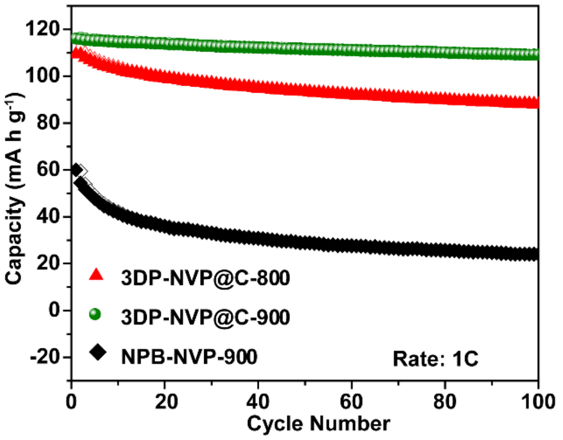

Figure S22. Cycling performances of 3DP-NVP@C-800, 3DP-NVP@C-900, and NPB-NVP-900.

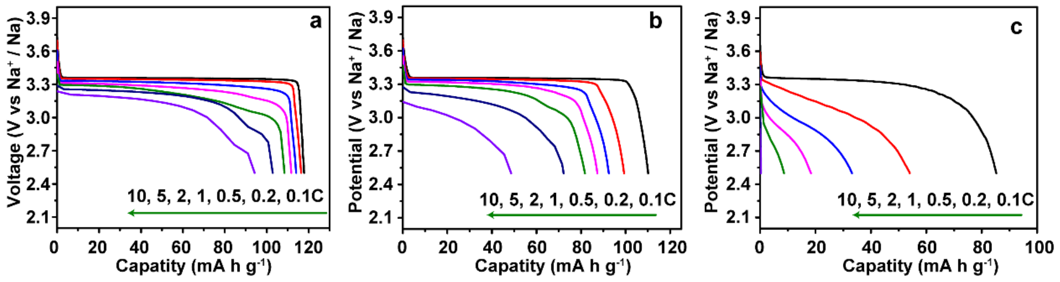

Figure S23. Discharge profiles of a) 3DP-NVP@C-900, b) 3DP-NVP@C-800 and c) NPB-NVP-900 at various current rates.

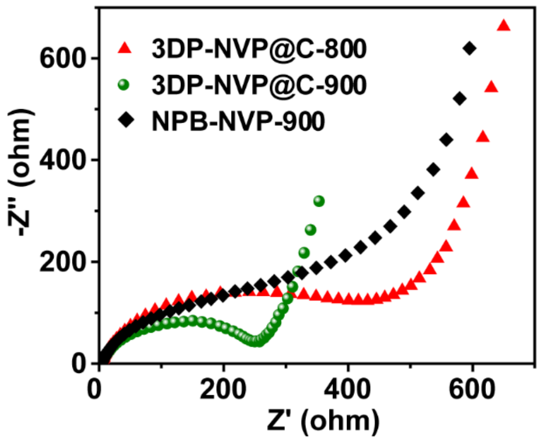

Figure S24. Nyquist plots of 3DP-NVP@C-800, 3DP-NVP@C-900, and NPB-NVP-900.

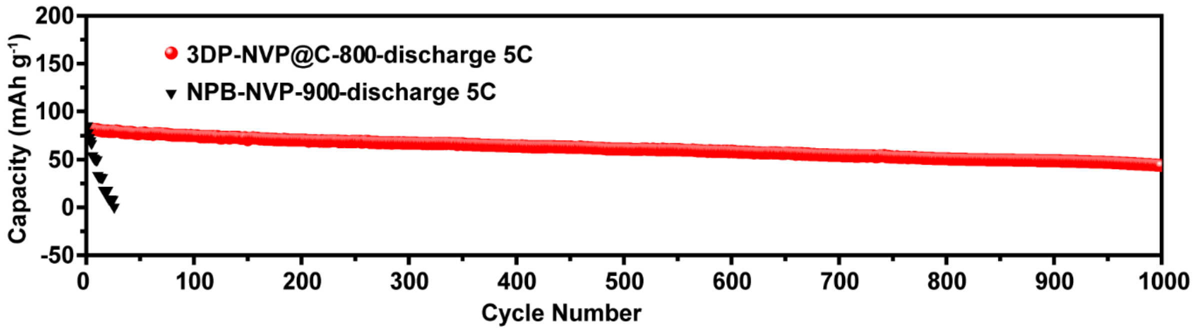

Figure S25. Long-term cycle life of 3DP-NVP@C-800 and NPB-NVP-900 for 1000 cycles at 5 C.

Table S1. Structural properties of the porous NVP.

Samples	Solvents	Polymer	BET surface area [m^2 g^{-1}]	Pore size [nm] Mesopores	Pore size [nm] Macropores	Pore volume [cm^3 g^{-1}]
3DP-NVP@C-700	THF/EtOH/H_2O	PEO-*b*-PS	77	10/30	100-500	0.272
3DP-NVP@C-800	THF/EtOH/H_2O	PEO-*b*-PS	51	10/30	100-500	0.205
3DP-NVP@C-900	THF/EtOH/H_2O	PEO-*b*-PS	20	10/30	100-500	0.151
NPB-NVP	H_2O	-	3	-	-	-
porous bulk NVP-1	EtOH/H_2O	-	13	-	100-2μm	0.0485
porous bulk NVP-2	THF/EtOH/H_2O	-	24	-	100-2μm	0.0805

Table S2. The detailed pore and throat parameters obtained by using pore network extraction method based on the maximal ball algorithm.

Number of pores	61255	Number of throats	156311
Maximum pore radius	0.3935	Maximum throat radius	0.35683
Average pore radius	0.12793	Average throat radius	0.06086
Maximum pore volume	7.95067	Maximum throat volume	0.23618
Average pore volume	0.136	Average throat volume	0.0059
Maximum coordination number	47	Maximum throat length	1.04518
Average coordination number	5.075	Average throat length	0.20211

Note: Volume [μm^3]; radius [μm].

Table S3. The binding energy (B.E.), relative peak area (P.A.) of C 1s, and percentage of C–C.

Samples	B.E. of C 1s (eV)				P.A. (Counts)				Percentage (%)
	C–C	C–O	C=O	O–C=O	C–C	C–O	C=O	O–C=O	C–C
3DP-NVP@C-700	283.7	284.3	285.1	287.5	77454	48191	30473	20552	43.8
3DP-NVP@C-800	284.0	284.7	286.0	288.6	69119	60094	16977	5730	45.4
3DP-NVP@C-900	284.0	284.7	286.0	288.6	69320	59629	18138	4931	45.6

Table S4. CHN elemental analysis of the 3DP-NVP@C obtained at different calcination temperatures and NPB-NVP.

Samples	Carbon contents
3DP-NVP@C-700	4.2%
3DP-NVP@C-800	2.1%
3DP-NVP@C-900	1.9%
NPB-NVP	0.1%

Table S5. Structural properties of porous NASICON-structured polyanion-type materials.

Samples	Solvents	Polymer	BET surface area [m^2 g^{-1}]	Pore volume [cm^3 g^{-1}]
$Li_3V_2(PO_4)_3$	THF/EtOH/H_2O	PEO-*b*-PS	53	0.204
$K_3V_2(PO_4)_3$	THF/EtOH/H_2O	PEO-*b*-PS	19	0.0879
$Na_4MnV(PO_4)_3$	THF/EtOH/H_2O	PEO-*b*-PS	18	0.0848
$Na_2TiV(PO_4)_3$	THF/EtOH/H_2O	PEO-*b*-PS	20	0.0937
$Na_3V_2(PO_4)_3$	THF/EtOH/H_2O	F127	30	0.0144
$Na_3V_2(PO_4)_3$	THF/EtOH/H_2O	P123	27	0.0132
$Na_3V_2(PO_4)_3$	THF/EtOH/H_2O	PEO-5000	62	0.222

Supplementary references

[S1] T. Gao, T. Wang, W. Wu, Y. Liu, Q. Huo, Z. Qiao, S. Dai, *Adv. Mater.* **2019**, *31*, 1806254.

[S2] F. Aranda, J. Hesserb, *Comput. Geosci.* **2017**, *101*, 28.

[S3] H. Dong, M. J. Blunt, *Phys. Rev. E* **2009**, *80*, 036307.

[S4] H. Sai, K. W. Tan, K. Hur, E. Asenath-Smith, R. Hovden, Y. Jiang, M. Riccio, D. A. Muller, V. Elser, L. A. Estroff, S. M. Gruner, U. Wiesner, *Science* **2013**, *341*, 530.

[S5] J. Hwang, C. Jo, K. Hur, J. Lim, S. Kim, J. Lee, *J. Am. Chem. Soc.* **2014**, *136*, 16066.

[S6] C. Jo, J. Hwang, W. Lim, J. Lim, K. Hur, J. Lee, *Adv. Mater.* **2018**, *30*, 1703829.

[S7] E. Wang, M. Chen, X. Liu, Y. Liu, H. Guo, Z. Wu, W. Xiang, B. Zhong, X. Guo, S. Chou, S. Dou, *Small Methods* **2019**, *3*, 1800169.
